# Epidemiology, Comorbidities and Associated Treatments, Therapeutic Management, and Clinical Outcomes in Patients with Prostate Cancer in Spain (SRealProstate): A Real-World Cohort Study

**DOI:** 10.3390/cancers18040554

**Published:** 2026-02-09

**Authors:** Angel Borque-Fernando, Nuria Romero-Laorden, Juan Francisco Rodríguez-Moreno, Noelia Alfaro-Oliver, Ariela Beliera-Kiendl, Elena Rebollo-Gómez, Ignacio Hernández, Jose Rubio-Briones

**Affiliations:** 1 Servicio de Urología, Hospital Universitario Miguel Servet, IIS-Aragón, 50009 Zaragoza, Spain; 2 Servicio de Oncología Médica, Hospital Universitario de La Princesa, UAM-Fundación Instituto Roche de Medicina Personalizada de Precisión, 28006 Madrid, Spain; nuriaromerolaorden@gmail.com; 3 Instituto de Investigación Sanitaria HM Hospitales, Facultad de Medicina, Universidad San Pablo CEU, 28003 Madrid, Spain; jf_rodriguezmoreno@hotmail.com; 4 Medical Department, AstraZeneca Farmacéutica Spain, 28050 Madrid, Spain; noelia.alfaro@astrazeneca.com (N.A.-O.); ariela.beliera@astrazeneca.com (A.B.-K.); 5 Atrys Health, 28002 Madrid, Spain; erebollo@atryshealth.com (E.R.-G.); ihernandez@atryshealth.com (I.H.); 6 Servicio de Urología, Hospital VITHAS 9 de Octubre, 46015 Valencia, Spain; jrubio@clinicadoctorrubio.es

**Keywords:** prostate cancer, prevalence, patient profile, treatment patterns, mCRPC, mHSPC, nmCRPC

## Abstract

**Background/Objectives**: Prostate cancer (PC) is the most prevalent cancer in men in Spain. Clinical management depends on the stage/tumor response to therapy/therapy availability. Given the limited national data, we analyzed real-world prevalence and management patterns. **Methods**: This was an observational, retrospective study using electronic medical records from public primary care centers/hospitals in Spain (BIG-PAC^®^ database), between 1 June 2014, and 31 December 2021. Adult PC-diagnosed patients were classified into localized PC with no compromised lymph nodes and no metastasis (N0/M0), locally advanced PC with compromised lymph nodes, no metastasis (N1/M0), metastatic hormone-sensitive PC (mHSPC), non-metastatic castration-resistant PC (nmCRPC), and metastatic castration-resistant PC (mCRPC, categorized by treatment line). Progression across stages was recorded. All analyses were descriptive and exploratory. **Results**: A total of 19,224 patients met the inclusion criteria. The five-year PC prevalence was 590 cases/100,000 males; localized PC was the most prevalent form of cancer (PC[N0/M0]: 473/100,000; PC[N1/M0]: 78/100,000), followed by mCRPC (16/100,000), mHSPC (14/100,000), and nmCRPC (8/100,000). We further analyzed 5583 patients with progression. Surgery was performed in 61.7% PC (N1/M0), while radiotherapy was used in 24.3%. Taxanes were used in 52.4% of the mHSPC patients. First prescription options for mCRPC L1 and L2 were androgen receptor pathway inhibitors (55.9% and 49.7%); 44.9% of mCRPC L3 and 83.3% of L4+ (≥4 treatment lines) patients used taxanes. Analgesics were common in mHSPC, nmCRPC, and mCRPC patients. Few mHSPC patients died without progression (11.6%); 90.2% and 56.2% of the mCRPC patients received first- and second-line treatments, respectively. During follow-up, 2436 patients died. Cardiovascular comorbidities increased with stage. **Conclusions**: PC management in Spain varies substantially by disease stage. Advanced disease was associated with higher comorbidity burden and reduced survival in mHSPC and mCRPC patients, despite multiple available treatments.

## Simple Summary

Prostate cancer represents the most common cancer among men in Spain, with treatment approaches and outcomes varying markedly by disease stage. Real-world data from 19,224 patients in Spain were analyzed to assess prostate cancer management across disease stages, and to examine its impact on survival and health status. Early-stage disease was most frequently managed with surgery or radiotherapy, whereas advanced stages required more intensive therapies and were associated with a higher rate of comorbidities such as cardiovascular disease. Despite the availability of treatments, patients with advanced prostate cancer continue to experience reduced survival and greater health challenges. These findings underscore the need for improved strategies to enhance patient management across all stages of the disease.

## 1. Introduction

Prostate cancer (PC) is the most prevalent cancer among Spanish men [1]. PC was the third leading cause of cancer deaths in men in 2021 in Spain (5889 deaths) and is estimated to be the most frequent new cancer diagnosed in men in 2025, with 32,188 new cases [1,2].

For early localized PC with no compromised lymph nodes and no metastasis (N0/M0), active surveillance and curative treatment strategies (radical prostatectomy, radiotherapy, and brachytherapy) are recommended [3,4]. Androgen deprivation therapy (ADT) with surgical/medical castration is used in advanced PC [3,5]. Treatments are intended to decrease tumor burden or avoid tumor growth. Although most patients’ prostate-specific antigen (PSA) levels decrease after ADT and are classified as hormone-sensitive PC (HSPC), the disease inevitably progresses to the non-curable castration-resistant phenotype (CRPC) [5].

PC may be classified according to the presence of distant metastasis, considered incurable [6]. In metastatic HSPC (mHSPC), ADT in combination with other treatments, such as androgen receptor pathway inhibitors (ARPIs) and systemic chemotherapy, has demonstrated efficacy [7,8]. The combination of ADT + ARPIs is the new standard, improving prognosis with a more favorable tolerability profile; some high-volume cases might be treated by adding chemotherapy to the ADT + ARPIs combination [3].

Metastatic CRPC (mCRPC) has a significantly poor prognosis and survival rate, despite the availability of treatments [3]. Treatment options have expanded in the last few decades, including ARPIs in many first-line settings [9] and radium-223 for bone metastases [10]. More recently, additional therapeutic strategies such as poly (ADP-ribose) polymerase (PARP) inhibitors and radioligand therapies (e.g., lutetium-177 [^177^Lu]) have further broadened the treatment landscape; however, these therapies were approved in Spain after the end of the study period and were therefore not available for evaluation in the present analysis, with PARP inhibitors obtaining approval only in 2025 [11,12,13,14,15,16,17,18]. Although substantial progress has been made, a consensus on the optimal treatment sequence beyond the second line has yet to be established [19,20].

Although guidelines are frequently updated based on clinical trials, PC is complex and clinically heterogeneous [3], and real-world evidence is required [21,22,23]. This retrospective study is the first linking national statistics with clinical data, calculating the real-world PC prevalence (overall and by stage [localized PC (N0/M0), locally advanced PC with compromised lymph nodes, no metastasis (N1/M0), mHSPC, nmCRPC, and mCRPC]) in Spain using the BIG-PAC^®^ database [24]. BIG-PAC^®^ contains more than 2.4 million electronic medical records (EMR) from users of the Spanish (public) health system. This study describes real-world prevalence, patient characteristics, treatment patterns, and clinical outcomes in adult men with PC in Spain, across five clinically defined stages.

## 2. Materials and Methods

### 2.1. Study Design, Data Source, and Population

This observational, retrospective study included adult males with a pathological report compatible with PC. The study period ranged from 1 June 2014 to 31 December 2021. The study design is presented in Figure S1.

The index date was the date of the first PC-positive result within the study period. This index date was subject to change according to the PC stage (as the disease progressed). Patients were followed until death or end of the study. Deaths recorded in the study were all-cause. The baseline period covered the 6 months prior to the index date.

The Research Ethics Committee of the Consorci Sanitari de Terrassa (Terrassa, Spain) approved this study protocol on 14 June 2022 (Code: 02-22-399-075). Patients’ consent to participate was not required.

Patients had to be enrolled in a medical prescription program, with records of daily dose, time interval, treatment duration, ≥2 prescriptions during the follow-up period (post-index date), and ≥2 health records in the computerized system.

Data were obtained from the BIG-PAC^®^ database, covering primary care centers and hospitals across seven Spanish regions (≈2.4 million EMR) [24] and from other associated hospitals within the same health areas. The BIG-PAC^®^ database has been shown to be representative of the Spanish adult population in terms of age, sex, and chronic disease burden in previous studies [25,26]. The database is registered with the European Medicines Agency (EMA) [24] and has been used in more than 40 studies over the last 5 years [27,28,29,30,31,32,33].

### 2.2. Patient Classification

Five cohorts were defined according to PC stage: localized PC (N0/M0), locally advanced PC (N1/M0), mHSPC, nmCRPC, and mCRPC. mCRPC was further categorized by treatment line (L): mCRPC first, second, third, and more than four lines (L1, L2, L3, and L4+, respectively).

Patients remained in their classification categories until the end of the follow-up period, death, or disease progression throughout any subsequent cohort. Consequently, patients could belong to multiple cohorts if their disease progressed.

For each patient, different key events with their index dates and baseline data were described: first diagnosis, first progression, second progression, etc.

Each treatment modification constituted a new line, except for changes in ADT. Treatment lines were defined based on therapies documented within the available data window. Although patient records were retrospectively reviewed from 1 January 2012, to ensure accurate cohort assignment, information prior to that date was unavailable; therefore, treatment sequences may not capture therapies received before database entry. As a result, L1 treatment for mCRPC reflects the first observed line within the database and may not represent the first lifetime systemic therapy for all patients. Consequently, treatment lines should be interpreted as observed sequences rather than complete lifetime treatment histories.

**Figure 1:**
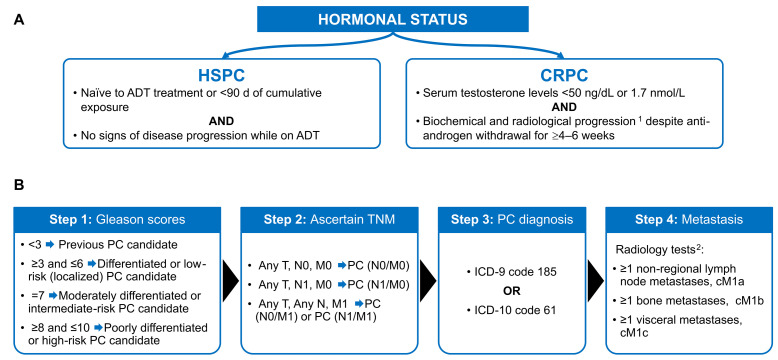
Definition for patient classification into study cohorts. (**A**) Definition of hormonal status (mCRPC patients were defined according to the Sociedad Española de Oncología Médica [SEOM] guidelines [2021] [34], based on the Prostate Cancer Working Group 2 criteria [35], and the European Association of Urology guidelines [2024]) [36]. (**B**) Steps for identifying patients with PC and their metastatic status from January 2012. ^1^ is as defined by the Prostate Cancer clinical Trials Working group 2. Biochemical progression included an increase in PSA levels, defined as 2 or more increases of ≥50% in successive determinations, and/or PSA levels > 2 ng/mL; ^2^ is including bone scan (prostate specific membrane antigen [PSMA] and positron emission tomography [PET]), computed tomography (CT) scan, and magnetic resonance imaging (MRI). ADT, androgen deprivation therapy; CRPC, castration-resistant prostate cancer; ICD-9, International Classification of Diseases Ninth revision; M#, metastasis or no metastasis; N#, compromised or not compromised lymph nodes; mHSPC, metastatic hormone-sensitive prostate cancer; nmCRPC, non-metastatic castration-resistant prostate cancer; PC, prostate cancer; T, tumor; TNM, tumor, regional lymph nodes, and distant metastasis status.

The algorithm for patients’ classification is shown in Figure 1. The PC N0/M0 stage was defined solely to calculate prevalence and to assess patient progression outcomes.

Modifications of treatment combinations implied progression, except for those with buserelin, leuprorelin, goserelin, triptorelin, and degarelix combinations.

Data regarding Gleason scores, tumor, node, and metastasis (TNM) status, and PC diagnosis based on International Classification of Diseases Ninth (ICD-9) or Tenth (ICD-10) revision (ICD-9 code 185 or ICD-10 code 61) [37,38] between 2012 and 2021 were considered to identify patients with a previous PC diagnosis. This approach ensured accurate assignment of patients to the corresponding study cohorts (Steps 1–4, Figure 1). Metastatic status was defined based on radiology tests and secondary tumor diagnoses (Figure 1).

For each stage, a new stage-specific index date was defined; this new index date was the date when the patient first fulfilled the criteria for that stage. Baseline characteristics were assessed in the 6 months preceding this stage-specific index date. Consequently, unique patients could contribute to more than one cohort if their disease progressed.

The denominators reported in stage-specific prevalence, and descriptive, and survival analyses correspond to ‘patient-stages’ (i.e., individuals within a particular stage) rather than unique patients. In contrast, the overall prevalence estimates and the analysis of progression between stages use unique patients as the unit of analysis (Table S1 provides a detailed breakdown). Readers should interpret patient numbers in each stage-specific analysis accordingly.

### 2.3. Objectives and Variables

The overall 5-year prevalence of PC in Spain, as well as stage-specific prevalence for the five defined categories (PC [N0/M0], PC [N1/M0], mHSPC, nmCRPC, and mCRPC), were estimated. For descriptive purposes, the number of patients alive on 31 December 2021 was reported to characterize the surviving population at the end of follow-up.

Additionally, the following variables were described for each clinical stage from PC (N1/M0) stage to mCRPC L4+ metastatic disease:

(1) Patients’ characteristics at baseline, including sociodemographic variables (age and body mass index [BMI]), habits (tobacco use and alcohol consumption), clinical variables (comorbidities and Charlson Comorbidity Index) [39], and treatments, including those for lower urinary tract symptoms and others.
(2) Clinical outcomes by PC clinical stage, including time of follow-up (a variable that describes the length of follow-up within each stage), survival during follow-up and causes of end of follow-up, overall survival (OS) (patients who advance to other stage or who were lost to follow-up were censored).
(3) Transitions through treatments and clinical parameters at the closest day after stage initiation or 15 days before progression to other stage (PC-related parameters: Gleason score, PSA, serum testosterone, alkaline phosphatase, hemoglobin, creatinine, glucose, and body weight).
(4) Therapeutic management of PC (i.e., treatments in each stage), and supportive treatments during the study period. In the case of PC (N1/M0), the database does not allow differentiation between surgery performed as part of the diagnostic process (after which a patient is classified as N1/M0) and surgery undertaken with therapeutic intent for a known N1/M0 condition. Therefore, surgical procedures are reported as exposure to surgery without inference about intent. PC treatments and concomitant medications were described according to the Anatomical Therapeutic Chemical (ATC) classification system and ICD-9/ICD-10 codes (Tables S2 and S3). Cardiovascular disease at baseline and during the study period (Table S4) and anemia grade III (hemoglobin < 10 g/dL) during the study period were defined using ICD-10 codes.

### 2.4. Statistical Analysis

Qualitative variables are presented as absolute and relative (%) frequencies; quantitative variables, as the mean and standard deviation (*SD*) and the median and 1st and 3rd quartiles (Q1, Q3, or interquartile range [*IQR*]). The 95% confidence interval (CI) was estimated. Five-year prevalence was defined as the number of men with a recorded diagnosis of PC who were alive at any time during 2014–2019 (numerator), divided by the total number of males alive in Spain during the same period (denominator), and expressed as cases per 100,000 individuals. The prevalence of each clinical stage was calculated as the percentage of all PC patients (the sum of PC [N0/M0], PC [N1/M0], mHSPC, nmCRPC, and mCRPC) on the same date. OS was estimated using the Kaplan–Meier estimator. Patients were censored if they progressed to the next disease stage. This approach was chosen to describe stage-specific survival within each cohort; however, it may underestimate overall mortality in more advanced stages and should be interpreted accordingly. Survival analyses were unadjusted, and no multivariable models were applied. SPSSWIN v.27 was used for all statistical analyses.

## 3. Results

### 3.1. Study Patients

Between 1 June 2014 and 31 December 2021, a total of 19,647 adult males were diagnosed with PC, of whom 19,224 met the inclusion criteria (Figure S2).

The five-year prevalence estimates were based on patients alive at any time during the 2014–2019 period: 13,641 localized PC (N0M0) patients, 1282 PC (N1/M0) patients, 244 mHSPC patients, 126 nmCRPC patients, and 307 mCRPC patients (Figure S2). For all subsequent analyses, PC (N0/M0) patients who did not progress (*n* = 13,641) were excluded.

The patients analyzed for further objectives with advanced PC comprised 5583 unique patients. Of those, 1736 patients were diagnosed initially with PC (N0/M0) and had at least one subsequent progression during follow-up (Table S1). The rest of the patients initiated the study at different stages.

Since we studied patients within stages (and patients may appear in more than one stage), overall, the numbers of patients analyzed in each cohort were 3560 PC (N1/M0) patients, 2062 mHSPC patients, 678 nmCRPC patients, and 2057 mCRPC patients (Table S1). As noted in the Methods, patients who progressed contributed to multiple cohorts with reassigned index dates; therefore, the stage-specific numbers represent patient-stages rather than unique individuals. Table S1 provides a summary of unique patient progression across stages.

### 3.2. Baseline Characteristics of Patients with Prostate Cancer

Table S1 and Figure S3 summarize the progression of unique patients through different disease stages. Table 1 presents the baseline characteristics of patients in the distinct PC stages 6 months before the index date. Overall, the patients’ characteristics were similar across groups; however, some increased as the disease progressed.

### 3.3. Prevalence of Prostate Cancer

The overall 5-year PC prevalence was 590 cases/100,000 males alive (*n* = 1,008,708). Most PC cases within the study were PC (N0/M0) (78.3%). PC (N1/M0) comprised 14.2%, whereas the other clinical stages amounted to <4% each (mCRPC was the most frequent of these) (Table 2).

The most prevalent stage was PC (N0/M0), with 473 cases/100,000 males, followed by PC (N1/M0) (78 cases/100,000 males) and mCRPC (16 cases/100,000 males) (Table 2).

### 3.4. Clinical Outcomes

#### 3.4.1. Overall Survival at Each Stage and Follow-Up

**Figure 2:**
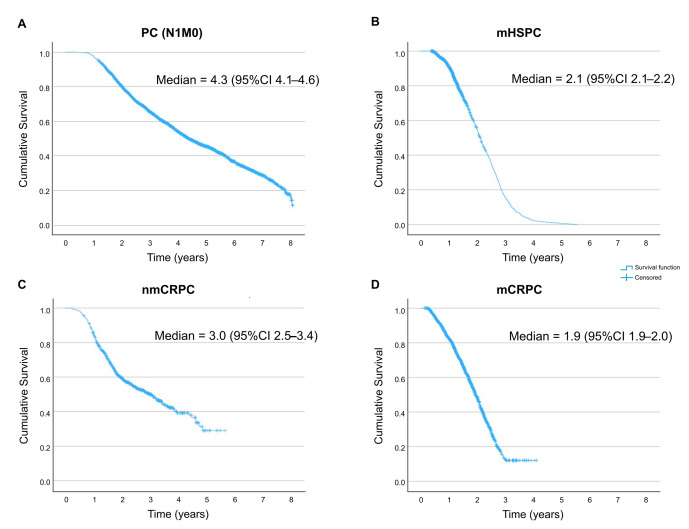
Kaplan–Meier curves for OS, censored at disease progression, in (**A**) PC (N1/M0), (**B**) mHSPC, (**C**) nmCRPC, and (**D**) mCRPC patients. CI, confidence interval; mHSPC, metastatic hormone-sensitive prostate cancer; nmCRPC, non-metastatic castration-resistant prostate cancer; mCRPC, metastatic castration-resistant prostate cancer; PC (N1/M0), prostate cancer with compromised lymph nodes, no metastasis.

PC (N1/M0) patients displayed a median OS of 4.3 (IQR, 5.0) years, reduced by half in the mHSPC stage and by almost one-third in the nmCRPC stages. Patients in the mCRPC stage had the lowest OS (1.9 [1.3] years) (Table 3, Figure 2).

**Figure 3:**
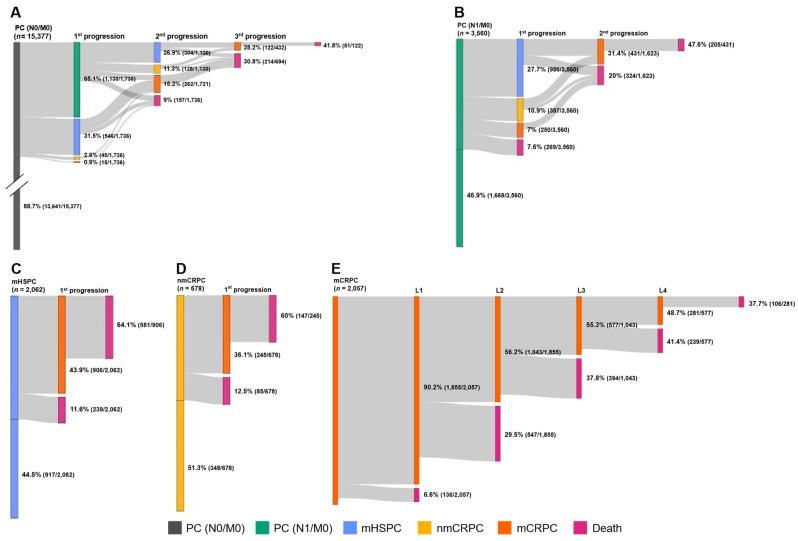
Transitions across the stage cohorts as they progress. Individual patients may appear in more than one cohort. (**A**) Cohort PC (N0/M0); (**B**) Cohort PC (N1/M0); (**C**) Cohort mHSPC; (**D**) Cohort nmCRPC; (**E**) mCRPC treatment lines. The lack of “grey flow” in some parts of the diagram represents patients who do not progress or whose study period has ended. mCRPC, metastatic castration-resistant prostate cancer; mCRPC, metastatic castration-resistant prostate cancer; mHSPC, metastatic hormone-sensitive prostate cancer; nmCRPC, non-metastatic castration-resistant prostate cancer; PC (N0/M0), prostate cancer (nearby lymph nodes 0/metastasis 0); PC (N1/M0), prostate cancer (nearby lymph nodes 1/metastasis 0).

The follow-up time was shorter for metastatic than for nmCRPC and PC (N1/M0) patients; most patients in PC (N1/M0), mHSPC, and nmCRPC stages survived (≥88.4%), contrasting with the 31.2% survival among mCRPC patients (Table 3). A total of 2009 patients died, most in the mCRPC stage (68.8%) (Table 3). The transition analysis of patients throughout stages and treatment lines showed a gradually increased proportion of patients dying and a gradually decreased proportion of patients progressing (Figure 3). Accordingly, the follow-up duration decreased as PC progressed. These results, along with the reasons for follow-up discontinuation are presented in Table 3.

Additionally, a subanalysis of mHSPC patients differentiating between metachronous mHSPC and de novo populations was performed (Table S5). In total, 530 mHSPC patients (2.8%) entered the study (unclassified in metachronous or de novo) (Table S1). These and those patients who progressed to mHSPC corresponded to 2062 patients (Tables S1 and S5). De novo mHSPC diagnosis occurred in 12.3% of the overall mHSPC population (Table S5). More metachronous mHSPC patients survived during follow-up than de novo patients in the mHSPC stage (90.8% vs. 71.5%) (Table S5).

#### 3.4.2. Clinical Parameters

**Figure 4:**
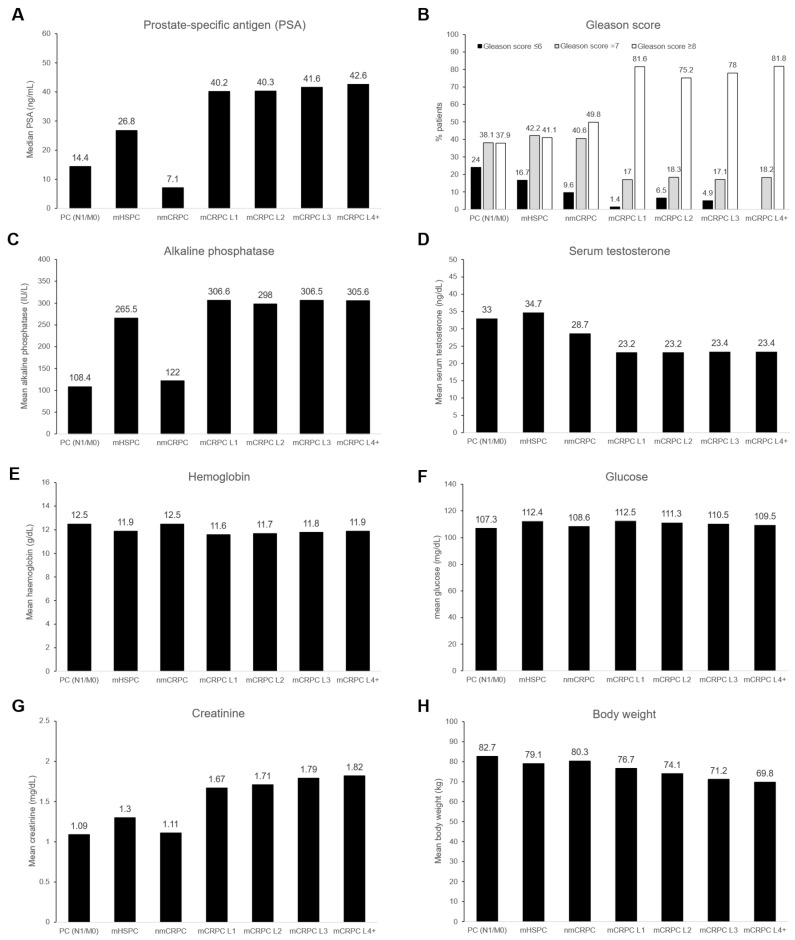
Clinical parameters according to prostate cancer clinical stage. (**A**) Prostate-specific antigen, (**B**) Gleason score, (**C**), alkaline phosphatase (**D**), serum testosterone, (**E**) hemoglobin, (**F**) glucose, (**G**) creatinine, and (**H**) body weight. Columns in (**B**) represent the % of patients according to Gleason scores and prostate cancer stage. Columns in (**A**,**C**–**H**) represent the mean. L, line; mCRPC, metastatic castration-resistant prostate cancer; mHSPC, metastatic hormone-sensitive prostate cancer; nmCRPC, non-metastatic castration-resistant prostate cancer; PC (N0/M0), prostate cancer (nearby lymph nodes 0/metastasis 0); PC (N1/M0), prostate cancer (nearby lymph nodes 1/metastasis 0).

All clinical parameters are found in Figure 4. The PSA values, Gleason score, and alkaline phosphatase (except for non-metastatic patients) increased as PC progressed, although to different extents (Figure 4A–C). Regarding blood parameters, hemoglobin and glucose were similar across PC stages (Figure 4E,F); creatinine was higher in the mCRPC patients (Figure 4F). Body weight modestly decreased throughout PC progression (Figure 4H).

#### 3.4.3. Cardiovascular Comorbidities

**Figure 5:**
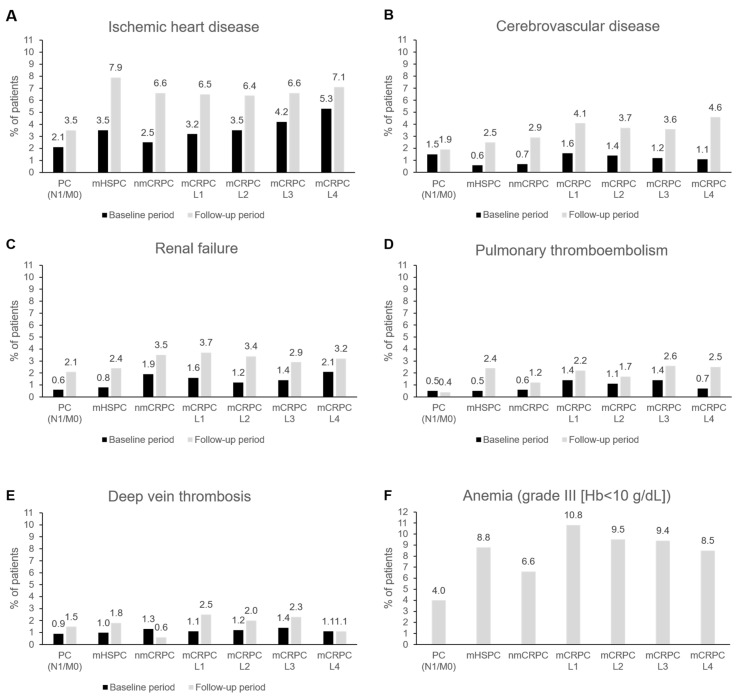
Proportion of patients with cardiovascular diseases at baseline and at follow-up ((**A**) Ischemic heart disease, (**B**) cerebrovascular disease, (**C**) renal failure, (**D**) Pulmonary thromboembolism, (**E**) deep vein thrombosis) and (**F**) proportion of patients with grade III anemia, according to prostate cancer clinical stage. Hb, hemoglobin; L, line; mHSPC, metastatic hormone-sensitive prostate cancer; nmCRPC, non-metastatic castration-resistant prostate cancer; mCRPC, metastatic castration-resistant prostate cancer; PC (N0/M0), prostate cancer (nearby lymph nodes 0/metastasis 0); PC (N1/M0), prostate cancer (nearby lymph nodes 1/metastasis 0).

The cardiovascular comorbidities are detailed in Figure 5. Patients with ischemic heart disease, cerebrovascular disease, and renal failure increased in every PC stage during follow-up compared to baseline; the difference between baseline and follow-up values was 0.4–4.4%. The sharpest difference was found for the mHSPC (Figure 5A), mCRPCL4 (Figure 5B), and mCRPCL2 patients (Figure 5C).

Differences were milder regarding pulmonary thromboembolism and deep vein thrombosis. Grade III anemia during follow-up was more frequent in the mCRPCL1, mCRPCL2, and mCRPCL3 patients (Figure 5F).

### 3.5. Therapeutic Management of PC Patients According to Disease Stage

**Figure 6:**
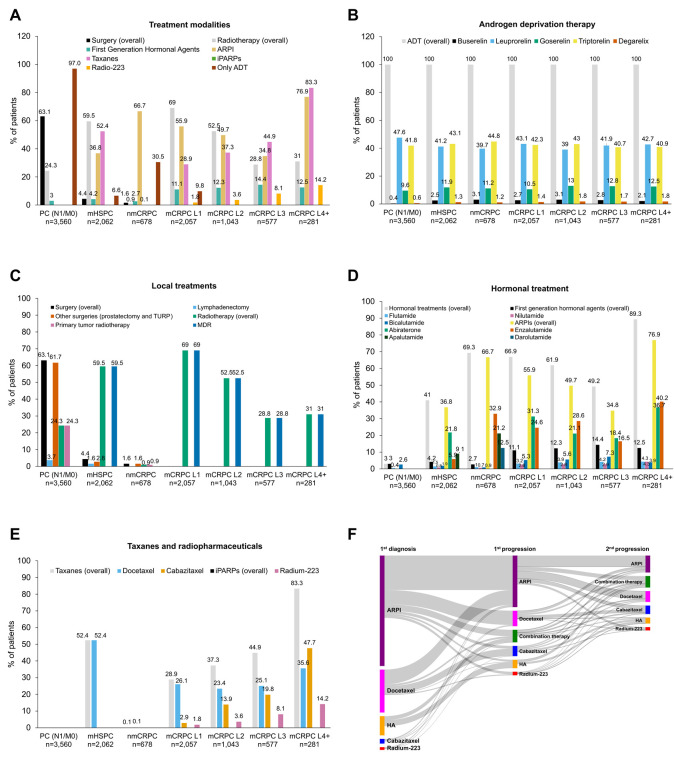
Prostate cancer treatments during the study period according to clinical stage. Values of 0 were removed from the labels to contribute to the graphic’s readability. (**A**) Overall treatment class. (**B**) Androgen deprivation therapy. (**C**) Surgery and radiotherapy. (**D**) Hormonal treatments. (**E**) Taxanes and radiopharmaceuticals; iPARPs (olaparib, niraparib, rucaparib, and talazoparib) were also recovered, but 0 results were retrieved in all stages. (**F**) Treatment sequencing from first diagnosis and throughout the first and second prostate cancer progression of study patients. The first diagnosis could be any stage of the disease, and would vary depending on the time of identification. ADT, androgen deprivation therapy; ARPIs, androgen receptor pathway inhibitors; HA, first-generation hormonal treatments; iPARPs, poly ADP ribose polymerase inhibitors; MDR, metastatic directed radiotherapy; mCRPC, metastatic castration-resistant prostate cancer; mHSPC, metastatic hormone-sensitive prostate cancer; nmCRPC, non-metastatic castration-resistant prostate cancer; PC (N0/M0), prostate cancer (nearby lymph nodes 0/metastasis 0); PC (N1/M0), prostate cancer (nearby lymph nodes 1/metastasis 0); TURP, transurethral resection of the prostate.

Overall, PC therapeutic management changed across disease stages (Figure 6A), except for ADT, which was used across all stages (Figure 6A,B). In the PC (N1/M0) patients, ADT was the only systemic therapy used in 97% of cases, with leuprorelin and triptorelin being the most frequently prescribed agents, while local treatments such as surgery and radiotherapy were also commonly administered (Figure 6B).

PC (N1/M0) patients were treated with surgery (63.1%) and radiotherapy (24.3%) (non-mutually exclusive), mainly for primary tumors (Figure 6C). Importantly, PC (N1/M0) surgeries aim (diagnostic/treatment) was undesignable (see Patients and Methods Sections).

Flutamide, nilutamide, and bicalutamide were used at low frequencies and similarly across disease stages, except in mCRPC (Figure 6D). The nmCRPC patients mostly received ARPIs as treatment (66.7%) (Figure 6D).

In the metastatic patients, the preferred treatments were chemotherapy, ARPIs, and radiotherapy (Figure 6C–E); regarding mCRPC, ARPIs were more frequent in L1 (55.9%) and L4+ (76.9%) patients. Of the 281 mCRPC L4+ patients, 83.3% and 14.2% received taxanes and radium-223, respectively.

Moreover, the use of ARPIs (Figure 6D) markedly differed among stages. The PC (N1/M0) patients did not receive ARPIs. The nmCRPC patients received enzalutamide (32.9%) and apalutamide (21.2%), and they were the only ones receiving darolutamide (12.5%). Abiraterone was preferred in the mHSPC patients (21.8%), followed by apalutamide (9.1%) and enzalutamide (5.9%). For the mCRPC patients, abiraterone was preferred in L1 (313%) and L2 (21.1%) patients, whereas enzalutamide was preferentially used in L3 (16.5%) and L4 (40.2%) patients (Figure 6D).

With respect to taxanes, the mHSPC patients only received docetaxel (52.4%), whereas 47.7% and 35.6% of the L4+ patients used cabazitaxel and docetaxel, respectively (Figure 6E). Radium-223 use increased throughout treatment lines (Figure 6E). The sequence of treatments across progressions is shown in Figure 6F. The complete set of data is included in Table S6.

Treatment duration (Table S7) progressively decreased, indicating a progressively faster progression throughout disease stages, and was markedly reduced upon progression to mCRPC. Besides PC treatments, patients received analgesic medications and antiresorptive bone agents (Table S8). mHSPC, nmCRPC, and mCRPC patients used more analgesics than PC (N1/M0) patients, whereas for antiresorptive bone agents, frequencies were higher in metastatic patients compared to PC (N1/M0) and nmCRPC patients.

## 4. Discussion

Here, we present real-world data from the users of the Spanish public health system with a pathological report compatible with PC between 2014 and 2021. A total of 5583 unique patients with localized or advanced PC were followed, many of whom progressed across disease stages over time. Analyses were conducted at the stage level on the different prevalences, overall survival, management, clinical parameters, and cardiovascular comorbidities. The study was designed as a descriptive, hypothesis-generating analysis. Accordingly, all comparisons across stages and treatment lines are unadjusted and should not be interpreted as causal or as evidence of treatment superiority.

The estimated five-year prevalence of PC in Spain was 590 cases per 100,000 males, consistent with previously reported national estimates [40]. As expected, localized PC (N0/M0) represented the most prevalent stage, followed by PC (N1/M0) and mCRPC. Differences in prevalence estimates across studies likely reflect variations in case definitions and methodological approaches. In the present study, the 5-year prevalence was the number of PC patients divided by the total population of Spain. Another study calculated the mCRPC prevalence as the number of people with a claim of mCRPC diagnosis in a given year, divided by all males in their database in the same year [41]. They found a prevalence of 9 cases/100,000 males in 2010 and 20 cases/100,000 in 2017, aligned with the 5-year prevalence of mCRPC of 16/100,000 males in our study.

The baseline patient characteristics were broadly similar across disease stages in terms of smoking status, alcohol use, hypertension, diabetes, and analgesic use. In contrast, the use of antiresorptive bone agents increased in the more advanced stages. Furthermore, OS was reduced in nmCRPC by one-third, and by half in mHSPC and mCRPC, compared to PC (N1/M0), despite the use of available treatments. Recent real-world evidence studies have displayed heterogeneous median OS values [42,43,44,45,46,47,48,49], underlining the complex scenario for mHSPC and mCRPC patients.

The treatment patterns varied according to disease stage and generally reflected contemporaneous clinical practice. Most PC (N1/M0) patients received surgery and/or radiotherapy (non-exclusive treatments), the majority of the nmCRPC patients used ARPIs (enzalutamide was the most frequent), and 52.5% of the mHSPC patients received docetaxel.

The mHSPC patients were treated with abiraterone, enzalutamide, apalutamide, and docetaxel in addition to ADT (treatment intensification), following guideline recommendations [34,50]. This contrasts with a recent study reporting that most patients do not receive these treatments, although their use was increasingly higher in Spain [51]. The EMA gave its positive opinion of abiraterone in 2017 [52], docetaxel in 2019 [53], apalutamide in 2019 [54], and enzalutamide in 2021 [55] for mHSPC patients. This study’s results (2014–2021) clearly reflect this sequential approval. Figure S4 summarizes the positive opinion and indication of each group in Spain.

In mCRPC, the preferred treatments were overall similar across the first and second lines. However, in the L3 and L4+ settings, ARPIs and taxanes were used similarly. Cabazitaxel and Radium-223 use was higher across the more advanced treatment lines in patients with mCRPC.

The treatment duration throughout PC progression was increasingly short. The treatment patterns observed in this real-world setting reflect, in general, the guideline recommendations up to 2021 (Table 4) [34,50]. However, advanced PC management is challenging, and regional differences in recommendations, treatment patterns, and availability have been documented [56]. The available treatments for mCRPC are palliative [57]. We found that 55.9% of the L1 patients used ARPIs (abiraterone and enzalutamide at similar frequencies) and 28.9% were treated with chemotherapy, similarly to previous reports [20,56]. Chemotherapy was the preferred treatment in L2 patients, and, in Spain, chemotherapy and ARPIs were equally used, in line with our results [56]. Radium-223 usage was scarce in mCRPC patients and only increased in L4+ (14.2%) cases. PARP inhibitors (iPARPs) were not used, as reflected in this study, likely due to a lack of funding from the Spanish healthcare system within the analyzed period. The treatment patterns observed during the study period should be interpreted in the context of evolving drug availability, reimbursement policies, and regulatory approvals in Spain. Apparent deviations from contemporaneous clinical guidelines may therefore reflect access limitations or delayed adoption rather than inappropriate clinical decision-making. Thus, it is not possible to fully disentangle the impact of evolving drug approvals, reimbursement policies, and guideline updates from true changes in physician preference over time; however, physician preference is limited by treatment indication and approval from the Spanish Agency of Medicines and Health Products (AEMPS). The Spanish healthcare system is tax-funded and universal. Therefore, observed temporal trends in treatment patterns should be interpreted as the net result of these intertwined factors rather than as pure indicators of prescriber choice.

Our study reaches 2021; however, issues regarding treatment controversies or lack of evidence are still ongoing, as the recent guidelines [56,57] and the conference on Advanced Prostate Cancer underlined [58]. Particularly, it is difficult to establish guidelines for the mCRPC stage, since the patients who reach this stage are very heterogeneous and their previous treatment and clinical history need to be taken into account [3,34,50,59]. In our study, ARPIs were preferred over taxanes in L1 (55.9% and 28.9%, respectively) and L2 (49.7% and 37.3%, respectively). In L3, taxanes were used over ARPIs (44.9% vs. 34.8%). Still, a preferred treatment for L4+ was missing, reflecting the lack of recommendations (the choice of therapy may be motivated by the patient’s toxicity profile) [19,21]. The observed use of ARPIs in later mCRPC lines should be interpreted in the context of real-world clinical constraints. During the study period, therapeutic options beyond the second or third line were limited, and treatment decisions in advanced mCRPC are frequently driven by patient frailty, tolerability, comorbidity burden, and prior treatment-related toxicities. Therefore, ARPIs use in L4+ likely reflects unmet clinical needs and feasibility considerations rather than expected therapeutic benefit, particularly in the presence of known cross-resistance mechanisms. More widespread use of ARPIs in mHSPC could impact the preference for taxanes for mCRPC over subsequent years. Cabazitaxel was increasingly used in advanced lines, likely following its indication after docetaxel failure [60], but only 11.4% of mCRPC patients received it across the different stages despite its demonstrated impact on prognosis [60], which may be related to their toxicity profile and patient clinical status in ≥L3. Moreover, treatment duration was increasingly shorter with each subsequent treatment line, likely due to cross-resistance development between ARPIs and taxanes, failing to achieve durable responses in very advanced patients, leaving place for new drugs with different mechanisms of action, such as iPARPs and Lutetium [57,61]. Despite treatment, most patients progressed or died. We have included Table 4 to compare scenarios to actual guidelines and perspectives over time.

ADT, ARPIs, and taxanes are associated with multiple side effects that require monitoring and concomitant treatments to mitigate symptoms. These include, among others, weight gain, increased cholesterol and triglyceride levels, insulin resistance, and bone mineral density loss, which impact cardiovascular function [62,63]. These patients frequently have baseline cardiovascular comorbidities, which PC treatments further worsen [64]. Our results showed increased cardiovascular comorbidities during follow-up for all PC stages. However, we cannot definitively attribute these increases to disease progression alone. Multiple competing mechanisms may be responsible of these increases, including (1) accumulating cardiovascular risk in an ageing cohort; (2) potential cardiometabolic effects of ADT and other therapies; (3) increased clinical surveillance and detection in advanced disease stages; and (4) disease progression itself. The observational design and available variables do not permit to quantify the relative contribution of each mechanism. Similarly, the increased frequency of grade III anemia in advanced mCRPC lines may reflect disease progression, bone marrow infiltration, cumulative effects of systemic therapies, or more frequent laboratory monitoring. Along these lines, though glucose levels were similar across PC stages, body weight decreased as the disease progressed. This finding could be related to cancer-associated cachexia, ADT-induced changes in body composition, treatment-related toxicity, or the cumulative effect of comorbidities [65]. However, the database does not capture direct measures of muscle mass or body composition, and therefore, sarcopenia cannot be formally assessed. These explanations remain hypothetical in our dataset.

This study has several limitations inherent to retrospective observational studies, including incomplete or missing information and the inability to infer causality. Only all-cause mortality was available, as cancer-specific death could not be retrieved. We retrospectively reviewed patient data 30 months before the study period to ensure the accurate classification of patients into study cohorts; however, this study’s results, particularly those regarding patient management, may not be generalizable to other settings (treatment utilization patterns depend on the organization and characteristics of healthcare systems and the drugs available for each indication in different countries). Moreover, treatment selection in mCRPC is strongly influenced by patient characteristics, disease trajectory, prior therapies, tolerability, and clinical judgment, which are not fully captured in routinely collected data. Therefore, confounding by indication cannot be excluded, and differences in treatment use across mCRPC lines should be interpreted as reflecting real-world practice rather than comparative effectiveness. Accordingly, treatment patterns are reported descriptively. In addition, treatment history prior to 1 January 2012 was unavailable. As a result, some patients classified as receiving first-line treatment for mCRPC may have received prior systemic therapies before their first recorded observation in the database. This limitation may bias treatment pattern descriptions and survival estimates by line, which should be interpreted as reflecting observed treatment sequences rather than complete lifetime treatment histories. Although the BIG-PAC^®^ database includes a large population from several autonomous regions, it does not cover all regions in Spain. Consequently, some degree of regional heterogeneity in treatment patterns and healthcare organization cannot be excluded. Nevertheless, PC management in Spain is delivered within a universal, tax-funded healthcare system and is largely guided by national and international clinical guidelines, supporting the relevance of the observed patterns as representative of routine clinical practice during the study period. Clinical guidelines and therapeutic indications have also evolved substantially over time, which may have influenced treatment distributions and should be considered when interpreting temporal trends. Finally, ambiguity regarding surgical intent in PC (N1/M0) may lead to misclassification of treatment strategies in this cohort; reported proportions of surgery should therefore be interpreted as descriptive of recorded procedures rather than definitive evidence of therapeutic intent.

Acknowledging these limitations, our aim was to provide a sound descriptive analysis to allow clear interpretation. This study provided valuable real-world data in Spain; to our knowledge, few studies have assessed the characteristics of mCRPC patients beyond the L3. Overall, this study’s results may be useful in optimizing the care of patients at different stages of advanced PC.

## 5. Conclusions

The PC (N0/M0) stage was the most prevalent in Spain, followed by PC (N1/M0) and mCRPC. As expected, advanced PC contributed to significant comorbidity burden and concomitant treatment use, increasing as PC progressed. Despite treatment guideline adherence and new treatment availability, the mHSPC and mCRPC patients’ OS remained short, and cardiovascular-related diseases still developed across the PC stage spectrum. We gathered vital information for patients in order to help them receive optimal treatment conditions and improve their well-being.

## Figures and Tables

**Table 1:** Baseline characteristics of advanced PC patients according to disease stage (PC [N0/M0] cohort is excluded).

	PC (N1/M0) *n* = 3560 ^†^	mHSPC *n* = 2062 ^†^	nmCRPC *n* = 678 ^†^	mCRPC L1 *n* = 2057 ^†^	mCRPC L2 *n* = 1043 ^†^	mCRPC L3 *n* = 577 ^†^	mCRPC L4+ *n* = 281 ^†^
**Sociodemographic characteristics**							
Age (years), mean (*SD*)	70.8 (7.9)	71.8 (7.9)	72.3 (7.4)	73.4 (7.9)	73.3 (7.9)	73.9 (7.7)	74.9 (7.8)
Age (years), ranges, *n* (%)							
18–44	1 (0)	0 (0)	0 (0)	0 (0)	0 (0)	0 (0)	0 (0)
45–54	71 (2.0)	39 (1.9)	4 (0.6)	31 (1.5)	13 (1.2)	3 (0.5)	0 (0)
55–64	718 (20.2)	352 (17.1)	106 (15.6)	279 (13.6)	153 (14.7)	82 (14.2)	29 (10.3)
65–74	1713 (48.1)	945 (45.8)	325 (47.9)	891 (43.3)	436 (41.8)	230 (39.9)	115 (40.9)
75–84	930 (26.1)	647 (31.4)	220 (32.4)	721 (35.1)	369 (35.4)	220 (38.1)	109 (38.8)
≥85	127 (3.6)	79 (3.8)	23 (3.4)	135 (6.6)	72 (6.9)	42 (7.3)	28 (10)
**BMI (kg/m^2^), mean (*SD*)**	28.5 (4.4)	28.3 (4.3)	28.3 (4.5)	27.5 (4.2)	25.0 (3.9)	24.0 (3.7)	23.5 (3.7)
**Habits, *n***	2391	144	260	276	276	276	276
Tobacco use, *n* (%)	256 (10.7)	18 (12.5)	32 (12.3)	30 (10.9)	30 (10.9)	30 (10.9)	30 (10.9)
Alcohol consumption, *n* (%)	94 (3.9)	10 (6.9)	19 (7.3)	11 (4.0)	11 (4.0)	11 (4.0)	11 (4.0)
**Associated comorbidities, *n* (%)**							
Hypertension	2093 (58.8)	1226 (59.5)	397 (58.6)	1217 (59.2)	616 (59.1)	352 (61)	169 (60.1)
Ischemic heart disease	410 (11.5)	288 (14.0)	82 (12.1)	268 (13.0)	128 (12.3)	72 (12.5)	35 (12.5)
Congestive heart failure	264 (7.4)	206 (10.0)	65 (9.6)	221 (10.7)	98 (9.4)	48 (8.3)	27 (9.6)
Peripheral vascular disease	293 (8.2)	216 (10.5)	75 (11.1)	225 (10.9)	98 (9.4)	55 (9.5)	25 (8.9)
Cerebrovascular disease	288 (8.1)	188 (9.1)	64 (9.4)	212 (10.3)	103 (9.9)	58 (10.1)	33 (11.7)
Dementia	84 (2.4)	80 (3.9)	28 (4.1)	91 (4.4)	37 (3.5)	25 (4.3)	12 (4.3)
Depression	270 (7.6)	189 (9.2)	68 (10.0)	212 (10.3)	102 (9.8)	56 (9.7)	24 (8.5)
Chronic pulmonary disease	447 (12.6)	331 (16.1)	88 (13.0)	334 (16.2)	162 (15.5)	96 (16.6)	50 (17.8)
Rheumatological disease	194 (5.4)	149 (7.2)	49 (7.2)	166 (8.1)	76 (7.3)	36 (6.2)	18 (6.4)
Peptic ulcer	212 (6.0)	159 (7.7)	56 (8.3)	173 (8.4)	66 (6.3)	38 (6.6)	17 (6.0)
Liver disease	221 (6.2)	154 (7.5)	47 (6.9)	163 (7.9)	72 (6.9)	40 (6.9)	18 (6.4)
Diabetes	927 (26)	605 (29.3)	189 (27.9)	611 (29.7)	297 (28.5)	160 (27.7)	78 (27.8)
Renal failure	332 (9.3)	222 (10.8)	78 (11.5)	228 (11.1)	107 (10.3)	57 (9.9)	20 (7.1)
Pulmonary thromboembolism	62 (1.7)	75 (3.6)	21 (3.1)	68 (3.3)	24 (2.3)	10 (1.7)	6 (2.1)
Deep vein thrombosis	83 (2.3)	80 (3.9)	21 (3.1)	78 (3.8)	30 (2.9)	20 (3.5)	14 (5)
AIDS	92 (2.6)	59 (2.9)	20 (2.9)	76 (3.7)	29 (2.8)	18 (3.1)	11 (3.9)
**General comorbidity (Charlson index), *n***	3560	2062	678	2057	1043	577	281
Charlson index, mean (*SD*)	2.1 (1.7)	2.5 (1.8)	2.4 (1.8)	2.6 (1.9)	2.3 (1.7)	2.4 (1.7)	2.4 (1.8)
Charlson index categories, *n* (%)							
0	459 (12.9)	180 (8.7)	67 (9.9)	163 (7.9)	101 (9.7)	57 (9.9)	28 (10.0)
1	1019 (28.6)	518 (25.1)	161 (23.7)	494 (24)	271 (26)	146 (25.3)	71 (25.3)
≥2	2082 (58.5)	1364 (66.1)	450 (66.4)	1400 (68.1)	671 (64.3)	374 (64.8)	182 (64.8)
**Previous treatments, *n***	3560	2062	678	2057	1043	577	281
For LUTS, *n* (%)							
Finasteride	70 (2.0)	63 (3.1)	18 (2.7)	72 (3.5)	25 (2.4)	18 (3.1)	11 (3.9)
Alfuzosin	72 (2.0)	85 (4.1)	20 (2.9)	73 (3.5)	26 (2.5)	15 (2.6)	5 (1.8)
Tamsulosin	862 (24.2)	522 (25.3)	177 (26.1)	531 (25.8)	261 (25.0)	140 (24.3)	56 (19.9)
Terazosin	45 (1.3)	60 (2.9)	22 (3.2)	57 (2.8)	29 (2.8)	16 (2.8)	6 (2.1)
Dutasteride	60 (1.7)	63 (3.1)	29 (4.3)	77 (3.7)	29 (2.8)	20 (3.5)	5 (1.8)
Other treatments, *n* (%)							
Statins	1688 (47.4)	1022 (49.6)	348 (51.3)	1060 (51.5)	548 (52.5)	302 (52.3)	144 (51.2)
Metformin	627 (17.6)	412 (20.0)	139 (20.5)	408 (19.8)	194 (18.6)	112 (19.4)	56 (19.9)
Acetylsalicylic acid	969 (27.2)	577 (28)	190 (28.0)	561 (27.3)	263 (25.2)	148 (25.6)	77 (27.4)
Analgesics	2322 (65.2)	1405 (68.1)	442 (65.2)	1422 (69.1)	743 (71.2)	413 (71.6)	202 (71.9)
Antiresorptive bone agents	275 (7.7)	441 (21.4)	181 (26.7)	440 (21.4)	225 (21.6)	123 (21.3)	61 (21.7)

^†^ Number of patients for all variables unless otherwise indicated. BMI, body mass index; mCRPC, metastatic castration-resistant prostate cancer; mHSPC, metastatic hormone-sensitive prostate cancer; L, treatment line; LUTS, lower urinary tract symptoms; nmCRPC, non-metastatic castration-resistant prostate cancer; PC (N1/M0), prostate cancer (nearby lymph nodes 1/metastasis 0); *SD*, standard deviation.

**Table 2:** Five-year prevalence of prostate cancer in Spain (2014–2019) and descriptive counts of survivors at the end of follow-up (31 December 2021).

	Local PC (N0/M0) *n* = 15,377	Locally Advanced PC (N1/M0) *n* = 3560	mHSPC *n* = 2062	nmCRPC *n* = 678	mCRPC *n* = 2057	Total PC—Including PC (N0/M0) *n* = 19,224
Alive within 2014–2019 (five-year prevalence numerator), *n*	4775	789	138	83	166	5951
Prevalence over males alive within 2014–2019 ^†^, cases/100,000 individuals	473	78	14	8	16	590
Alive on 31 December 2021, *n* ^‡^	7070	1282	244	126	307	9029
Proportion of patients diagnosed with PC and alive on 31 December 2021, %	78.3	14.2	2.7	1.4	3.4	100

^†^ Total of 1,008,708 males alive within 2014–2019. mCRPC, metastatic castration-resistant prostate cancer. ^‡^ Counts of patients alive on 31 December 2021 are provided for descriptive purposes and do not correspond to the five-year prevalence definition. mHSPC, metastatic hormone-sensitive prostate cancer; nmCRPC, non-metastatic castration-resistant prostate cancer; PC (N0/M0), prostate cancer (nearby lymph nodes 0/metastasis 0); PC (N1/M0), prostate cancer (nearby lymph nodes 1/metastasis 0).

**Table 3:** Follow-up time, cause of end of follow-up, survival in the follow-up period, and OS of each stage of PC.

	PC (N1/M0)	mHSPC	nmCRPC	mCRPC (Overall)	mCRPC L1	mCRPC L2	mCRPC L3	mCRPC L4+
*n* (%)	3560 (100)	2062 (100)	678 (100)	2057 (100)	2057 (100)	1043 (100)	577 (100)	281 (100)
**Follow-up duration (years)**								
Mean (*SD*)	4.3 (2.6)	1.4 (0.7)	1.9 (0.8)	0.9 (0.2)	0.5 (0.2)	0.4 (0.1)	0.4 (0.2)	0.4 (0.2)
Median (*IQR*)	2.8 (2–7.6)	1 (0.8–2.0)	1.9 (1.2–2.6)	1.1 (0.9–1.4)	0.4 (0.3–0.7)	0.5 (0.4–0.5)	0.4 (0.3–0.4)	0.4 (0.3–0.6)
Survival in the follow-up period, *n* (%)	3291 (92.4)	1823 (88.4)	593 (87.5)	641 (31.2)	1045 (50.8)	649 (62.2)	338 (58.6)	175 (62.3)
**Reasons to end patient’s follow-up *n* (%)**								
All-cause death	269 (7.6)	239 (11.6)	85 (12.5)	1416 (68.8)	677 (32.9)	394 (37.8)	239 (41.4)	106 (37.7)
Disease progression	1623 (45.6)	906 (43.9)	245 (36.1)	0	1043 (50.7)	577 (55.3)	281 (48.7)	0 (0)
End of study	1621 (45.5)	819 (39.7)	332 (49.0)	547 (26.6)	318 (15.5)	41 (3.9)	38 (6.6)	150 (53.4)
Loss to follow-up	47 (1.3)	98 (4.8)	16 (2.4)	94 (4.6)	19 (0.9)	31 (3.0)	19 (3.3)	25 (8.9)
OS at each stage ^†^ (years, median, *IQR*)	4.3 (5.0)	2.1 (1.3)	3.0 (1.3)	1.9 (1.3) ^‡^	-	-	-	-

^†^ Patients were censored upon progression to other stage; ^‡^ OS was calculated for the whole mCRPC period. mCRPC, metastatic castration-resistant prostate cancer; mHSPC, metastatic hormone-sensitive prostate cancer; *IQR*, interquartile range; L, treatment line; nmCRPC, non-metastatic castration-resistant prostate cancer; PC (N1/M0), prostate cancer (nearby lymph nodes 1/metastasis 0); OS, overall survival; *SD*, standard deviation.

**Table 4:** Most-used treatments or approaches in our study and treatment perspective overview across the years, according to clinical guidelines.

	Results from the Study	2021 Clinical Guidelines ^‡^	2023–2025 Clinical Guidelines ^§,⁋^
**PC (N1/M0)**	ADT + surgery and/or RT (non-exclusive treatments)	RP +/− pelvic lymphadenectomy ADT + RT	RT + 2–3 years ADT + 2 years abiraterone RP
**mHSPC**	ADT + RT ^†^ ADT + abiraterone ADT + enzalutamide ADT + apalutamide ADT + docetaxel ^†^	ADT + abiraterone ADT + docetaxel ADT + apalutamide ADT + enzalutamide ADT + docetaxel + RT	ADT + docetaxel +/− abiraterone/darolutamide (triplet therapy indicated only for high risk) ADT + RT to the primary tumor + ARPI ADT + abiraterone/apalutamide/enzalutamide/darolutamide
**nmCRPC**	ADT + enzalutamide ADT + darolutamide ADT + apalutamide	ADT + enzalutamide ADT + darolutamide ADT + apalutamide	ADT + enzalutamide ADT + darolutamide ADT + apalutamide
**mCRPC L1**	ADT + RT ^†^ ADT + abiraterone ADT + enzalutamide ADT + docetaxel ^†^	1st docetaxel → 2nd cabazitaxel ADT + abiraterone ADT + enzalutamide	1st docetaxel → 2nd cabazitaxel ADT+ Abiraterone +/− olaparib/niraparib ADT+ Enzalutamide +/− talazoparib ^117^Lu-PSMA
**mCRPC L2**	ADT + RT ^†^ ADT + abiraterone ADT + enzalutamide ADT + docetaxel ^†^ ADT + cabazitaxel	Cabazitaxel ADT + abiraterone ADT + enzalutamide Docetaxel	Cabazitaxel ADT+ Abiraterone ADT+ Enzalutamide ^223^Ra Cabazitaxel/^223^Ra/^117^Lu-PSMA-617/ olaparib PARP inhibitor Docetaxel rechallenge ADT+ ARPI followed by PARP inhibitor
**mCRPC L3**	ADT + docetaxel ^†^ ADT + cabazitaxel ADT + abiraterone ADT + enzalutamide ADT + RT ^†^	Cabazitaxel 223-Ra	Not specified Cabazitaxel
**mCRPC L4+**	ADT + cabazitaxel ADT + docetaxel ^†^ ADT + enzalutamide ADT + abiraterone ADT + RT ^†^	Not specified	Not specified

^†^ Our analysis could not discern if a treatment was given together or before/after any combination; ^‡^ [34,50]; ^§^ [3,58,59]; ^⁋^ all the options below are not suitable for all patients, and restrictions apply; please consult each clinical guideline for further specification. ADT, androgen deprivation therapy; mCRPC, metastatic castration-resistant prostate cancer; mHSPC, metastatic hormone-sensitive prostate cancer; ^117^Lu-PSMA-617, lutetium-177 combined with prostate-specific membrane antigen inhibitor; nmCRPC, non-metastatic castration-resistant prostate cancer; PC (N0/M0), prostate cancer (nearby lymph nodes 0/metastasis 0); PC (N1/M0), prostate cancer (nearby lymph nodes 1/metastasis 0); ^223^Ra, radium-223; RP, radical prostatectomy; RT, radiotherapy.

## Data Availability

The datasets generated and analyzed during the current study are not available from the corresponding author because they include patient data.

## Supplementary Materials

The following supporting information can be downloaded at https://www.mdpi.com/article/10.3390/cancers18040554/s1: Table S1: Progression of patients throughout prostate cancer stages during the study; Table S2: Anatomical Therapeutic Chemical (ATC) and International Classification of Diseases (ICD) codes for prostate cancer treatments; Table S3: ATC and ICD-10 codes for previous and concomitant treatments; Table S4: ICD-10 codes for additional evaluations; Table S5: Follow-up in mHSPC patients; Table S6: Prostate cancer patients who undergo treatments during the follow-up period according to disease stage, *n* (%); Table S7: Duration of prostate cancer treatments during the follow-up period according to disease stage; Table S8: Concomitant medication and procedures during the follow-up period; Figure S1: Diagram of the study design; Figure S2: Patient selection flow chart and classification into study cohorts; Figure S3: Flow of unique patients from the moment they entered the study until the end of study or follow-up; Figure S4: Financial situation and therapeutic positioning reports/technical sheets or national clinical guidelines.

## Abbreviations

The following abbreviations are used in this manuscript:

ADT	Androgen deprivation therapy
ARPI	Androgen receptor pathway inhibitor
ATC	Anatomical therapeutic chemical classification
BMI	Body mass index
CI	Confidence interval
CRPC	Castration-resistant prostate cancer
EAU	European Association of Urology
EMA	European Medicines Agency
EMR	Electronic medical records
ESMO	European Society for Medical Oncology
HSPC	Hormone-sensitive prostate cancer
ICD	International Classification of Diseases
IQR	Interquartile range
L	Line (treatment line)
m	Metastatic
mCRPC	Metastatic castration-resistant prostate cancer
mHSPC	Metastatic hormone-sensitive prostate cancer
nm	Non-metastatic
nmCRPC	Non-metastatic castration-resistant prostate cancer
OS	Overall survival
PC	Prostate cancer
PSA	Prostate-specific antigen
Q1	First quartile
Q3	Third quartile
RECORD	REporting of studies Conducted using Observational Routinely-collected health Data
RT	Radiotherapy
SD	Standard deviation
SEOM	Sociedad Española de Oncología Médica
T	Tumor
TNM	Tumor, node, and metastasis classification
TURP	Transurethral resection of the prostate
