# Supplementary material for: Epidemiology, Comorbidities and Associated Treatments, Therapeutic Management, and Clinical Outcomes in Patients with Prostate Cancer in Spain (SRealProstate): A Real-World Cohort Study
Source: Cancers (Basel). 2026 Feb 9;18(4):554. doi: 10.3390/cancers18040554 (PMC12938519; doi:10.3390/cancers18040554)
Supplement: Supplementary file 1 [file cancers-18-00554-s001.zip › cancers-4100870-supplementary.pdf]

Supplementary Materials

Epidemiology, Comorbidities and associated treatments, Therapeutic Management, and Clinical Outcomes in Patients with Prostate Cancer in Spain (SRealProstate): A Real–world Cohort Study

Supplementary Tables

Table S1. Progression of patients throughout prostate cancer stages during the study. 2

Table S2. Anatomical Therapeutic Chemical (ATC) and International Classification of Diseases (ICD) codes for prostate cancer treatments. 3

Table S3. ATC and ICD–10 codes for previous and concomitant treatments. 4

Table S4. ICD–10 codes for additional evaluations. 4

Table S5. Follow–up in mHSPC patients 5

Table S6. Prostate cancer patients who undergo treatments during the follow–up period according to disease stage, *n (%)*. 6

Table S7. Duration of prostate cancer treatments during the follow–up period according to disease stage. 7

Table S8. Concomitant medication and procedures during the follow–up period. 9

Supplementary figures

Figure S1. Diagram of the study design. 10

Figure S2. Patient selection flow chart and classification into study cohorts. 11

Figure S3. Flow of unique patients from the moment they entered the study until the end of the study or follow–up. 12

Figure S4. Financial situation and therapeutic positioning reports/technical sheets or national clinical guidelines. 13

Supplementary Tables

Table S1. Progression of patients throughout prostate cancer stages during the study.

1st stage	2nd stage	3rd stage	4th stage	Number. of unique patients	Percentage	Number of patients				
						PC (N0/M0)	PC (N1/M0)	mHSPC	nmCRPC	mCRPC
PC (N0/M0)	–	–	–	13,641†	70.96%	13,641				
PC (N0/M0)	PC (N1/M0)	–	–	622	3.24%	622	622			
PC (N0/M0)	mHSPC	–	–	377	1.96%	377		377		
PC (N0/M0)	PC (N1/M0)	mHSPC	–	223	1.16%	223	223	223		
PC (N0/M0)	mHSPC	mCRPC	–	169	0.88%	169		169		169
PC (N0/M0)	PC (N1/M0)	nmCRPC	–	87	0.45%	87	87		87	
PC (N0/M0)	PC (N1/M0)	mHSPC	mCRPC	81	0.42%	81	81	81		81
PC (N0/M0)	PC (N1/M0)	mCRPC	–	76	0.40%	76	76			76
PC (N0/M0)	PC (N1/M0)	nmCRPC	mCRPC	41	0.21%	41	41		41	41
PC (N0/M0)	nmCRPC	mCRPC	–	17	0.09%	17			17	17
PC (N0/M0)	nmCRPC	–	–	28	0.15%	28			28	
PC (N0/M0)	mCRPC	–	–	15	0.08%	15				15
PC (N1/M0)	–	–	–	1,315	6.84%		1,315			
PC (N1/M0)	mHSPC	–	–	474	2.47%		474	474		
PC (N1/M0)	mHSPC	mCRPC	–	208	1.08%		208	208		208
PC (N1/M0)	mCRPC	–	–	174	0.91%		174			174
PC (N1/M0)	nmCRPC	–	–	158	0.82%		158		158	
PC (N1/M0)	nmCRPC	mCRPC	–	101	0.53%		101		101	101
mHSPC	mCRPC	–	–	448	2.33%			448		448
mHSPC	–	–	–	82	0.43%			82		
nmCRPC	–	–	–	160	0.83%				160	
nmCRPC	mCRPC	–	–	86	0.45%				86	86
mCRPC	–	–	–	641	3.33%					641
Total unique patients / total patients within stage				19,224‡<br>5,583§ (without N0M0)	100.00%	15,377¶	3,560¶	2,062¶	678¶	2,057¶

†Considered only for the calculation of the overall prostate cancer prevalence.
‡Population for the first objective.
§Population for the secondary objectives (all types except those PC [N0M0] who did not progress during the study period).
¶These numbers are the sum of the patients within each cohort. Please beware patients may be part of more than one cohort (as they progress).
M#, metastasis o no metastasis; N#/, compromised or not lymph nodes; mHSPC, metastatic hormone–sensitive prostate cancer; nmCRPC, non–metastatic castration–resistant prostate cancer; mCRPC, metastatic castration–resistant prostate cancer; PC, prostate cancer; TNM, tumor, node and metastasis.

Table S2. Anatomical Therapeutic Chemical (ATC) and International Classification of Diseases (ICD) codes for prostate cancer treatments.

**Treatments**	**Codes**
**Surgeries for primary prostate tumor**	
Lymphadenectomy	07B00ZZ–07BJ4ZZ and 07T00ZZ–07TJ4ZZ
Other surgeries	
Prostatectomy	0VT00ZZ, 0VT04ZZ, 0VT07ZZ, 0VT08ZZ, 0VB00ZZ, 0VB03ZZ, 0VB04ZZ, 0V500ZZ, 0V503ZZ, 0V504ZZ,
Transurethral resection of the prostate	0V507ZZ, 0V508ZZ, 0VB07ZZ, 0VB08ZZ, 0VT07ZZ, 0VT08ZZ
**Taxanes**	
Docetaxel	L01CD02
Cabazitaxel	L01CD04
**iPARPs**	
Olaparib	L01XK01
Niraparib	L01XK02
Rucaparib	L01XK03
Talazoparib	L01XK04
**Androgen deprivation therapy**	
Buserelin[†]	L02AE01
Leuprorelin[†]	L02AE02
Goserelin[†]	L02AE03
Triptorelin[†]	L02AE04
Degarelix[†]	L02BX02
**First generation hormonal agents**	
Flutamide	L02BB01
Nilutamide	L02BB02
Bicalutamide	L02BB03
**New hormonal agents**	
Abiraterone	L02BX03
Enzalutamide	L02BB04
Apalutamide	L02BB05
Darolutamide	L02BB06
**Radiopharmaceuticals**	
Radio–223 (^{223}Ra)	V10XX03

[†]drugs usually maintained as part of the ADT combinations despite the disease progression

Table S3. ATC and ICD–10 codes for previous and concomitant treatments.

Treatments	Codes
**Previous treatments**	
**For lower urinary tract symptoms**	
Finasteride	G04CB01
Dutasteride	G04CB02
Alfuzosin	G04CA01
Tamsulosin	G04CA02
Terazosin	G04CA03
**Other treatments**	
Statins	C10AA and C10BA
Metformin	A10BA02
Acetylsalicylic acid	B01AC06 and N02BA01
**Concomitant medications**	
Analgesic and pain medications	N02
Antiresorptive bone agents	M5B
**Blood and other transfusions**	30233H0, 30233N0, 30243H0, 30243N0, 30253H0, 30253N0, 30263H0 and 30263H0

Table S4. ICD–10 codes for additional evaluations

Disease	Codes
Cardiovascular diseases	
Ischemic heart disease	I20–I25
Cerebrovascular diseases	G04CB01
Pulmonary thromboembolism	G04CB02
Renal failure	N17–N19
Deep vein thrombosis	I82.409

Table S5. Follow–up in mHSPC patients

Study groups	PC (N1/M0)	mHSPC	nmCRPC	mCRPC L1	mCRPC L2	mCRPC L3	mCRPC L4+
**All mHSPC**							
N (%)	986 (100)	2,062 (100)	0 (100)	906 (100)	452 (100)	262 (100)	137 (100)
Duration of follow–up period (years)							
Mean (*SD*)	3.2 (2.4)	1.4 (0.7)	0 (–)	0.5 (0.2)	0.4 (0.1)	0.4 (0.2)	0.4 (0.2)
Median (*IQR*)	1.7 (1.4)	1 (0.8)	0 (–)	0.4 (0.3)	0.5 (0.4)	0.4 (0.3)	0.4 (0.3)
Survival during follow–up (*N*, %)	986 (100)	1,823 (88.4)	0 (–)	454 (50.1)	303 (67)	163 (62.2)	90 (65.7)
Reasons to end patient's follow–up (*N*, %)							
All–cause death	0 (0)	239 (11.6)	0 (–)	452 (49.9)	149 (33)	99 (37.8)	47 (34.3)
Disease progression†	986 (100)	906 (43.9)	0 (–)	0 (0)	262 (58)	137 (52.3)	0 (0)
End of study	0 (0)	819 (39.7)	0 (–)	349 (38.5)	30 (6.6)	16 (6.1)	80 (58.4)
Loss of follow–up	0 (0)	98 (4.8)	0 (–)	105 (11.6)	11 (2.4)	10 (3.8)	10 (7.3)
**Metachronous**							
N, %	986 (100)	1,809 (100)	0 (–)	847 (100)	416 (100)	243 (100)	125 (100)
Duration of follow–up period (years)							
Mean (*SD*)	3.2 (2.4)	1.5 (0.7)	0 (–)	0.5 (0.2)	0.4 (0.1)	0.4 (0.2)	0.4 (0.2)
Median (*IQR*)	1.7 (1.4)	1.2 (0.8)	0 (–)	0.4 (0.3)	0.5 (0.4)	0.4 (0.3)	0.4 (0.3)
Survival during follow–up (*N*, %)	986 (100)	1,642 (90.8)	0 (–)	429 (50.6)	282 (67.8)	151 (62.1)	81 (64.8)
Reasons to end patient's follow–up (*N*, %)							
All–cause death	0 (0)	167 (9.2)	0 (–)	418 (49.4)	134 (32.2)	92 (37.9)	44 (35.2)
Disease progression†	986 (100)	847 (46.8)	0 (–)	0 (0)	243 (58.4)	125 (51.4)	0 (0)
End of study	0 (0)	708 (39.1)	0 (–)	332 (39.2)	28 (6.7)	16 (6.6)	73 (58.4)
Loss of follow–up	0 (0)	87 (4.8)	0 (–)	97 (11.5)	11 (2.6)	10 (4.1)	8 (6.4)
De novo							
N, %	0 (–)	253 (100)	0 (–)	59 (100)	36 (100)	19 (100)	12 (100)
Duration of follow–up period (years)							
Mean (*SD*)	0 (–)	1.1 (0.5)	0 (–)	0.5 (0.2)	0.5 (0.1)	0.4 (0.1)	0.4 (0.2)
Median (*IQR*)	0 (–)	0.9 (0.8)	0 (–)	0.4 (0.3)	0.5 (0.4)	0.4 (0.3)	0.4 (0.3)
Survival during follow–up (*N*, %)	0 (–)	181 (71.5)	0 (–)	25 (42.4)	21 (58.3)	12 (63.2)	9 (75)
Reasons to end patient's follow–up (*N*, %)							
All–cause death	0 (–)	72 (28.5)	0 (–)	34 (57.6)	15 (41.7)	7 (36.8)	3 (25)
Disease progression†	0 (–)	59 (23.3)	0 (–)	0 (0)	19 (52.8)	12 (63.2)	0 (0)
End of study	0 (–)	111 (43.9)	0 (–)	17 (28.8)	2 (5.6)	0 (0)	7 (58.3)
Loss of follow–up	0 (–)	11 (4.3)	0 (–)	8 (13.6)	0 (0)	0 (0)	2 (16.7)

†disease progression does not include death. M#, metastasis o no metastasis; N#/, compromised or not lymph nodes; mHSPC, metastatic hormone–sensitive prostate cancer; nmCRPC, non–metastatic castration–resistant prostate cancer; mCRPC, metastatic castration–resistant prostate cancer; PC, prostate cancer.

Table S6. Prostate cancer patients who undergo treatments during the follow–up period according to disease stage, *n (%).*

	PC (N1/M0) n=3560	mHSPC n=2062	nmCRPC n=678	mCRPC L1 n=2057	mCRPC L2 n=1043	mCRPC L3 n=577	mCRPC L4+ n=281
**Surgeries (overall)**	**2248 (63.1)**	**90 (4.4)**	**11 (1.6)**	**0 (0)**	**0 (0)**	**0 (0)**	**0 (0)**
Other surgeries†	2196 (61.7)	58 (2.8)	11 (1.6)	0 (0)	0 (0)	0 (0)	0 (0)
Lymphadenectomy	130 (3.7)	32 (1.6)	0 (0)	0 (0)	0 (0)	0 (0)	0 (0)
**Radiotherapy (overall)**	**864 (24.3)**	**1227 (59.5)**	**6 (0.9)**	**1420 (69)**	**548 (52.5)**	**166 (28.8)**	**87 (31)**
Primary tumor radiotherapy	864 (24.3)	0 (0)	6 (0.9)	0 (0)	0 (0)	0 (0)	0 (0)
MDR	0 (0)	1227 (59.5)	0 (0)	1420 (69)	548 (52.5)	166 (28.8)	87 (31)
**Medications**							
ADT	**3560 (100)**	**2062 (100)**	**678 (100)**	**2057 (100)**	**1043 (100)**	**577 (100)**	**281 (100)**
Buserelin*	15 (0.4)	51 (2.5)	21 (3.1)	56 (2.7)	32 (3.1)	16 (2.8)	6 (2.1)
Leuprorelin*	1694 (47.6)	850 (41.2)	269 (39.7)	887 (43.1)	407 (39)	242 (41.9)	120 (42.7)
Goserelin*	343 (9.6)	246 (11.9)	76 (11.2)	215 (10.5)	136 (13)	74 (12.8)	35 (12.5)
Triptorelin*	1488 (41.8)	888 (43.1)	304 (44.8)	871 (42.3)	449 (43)	235 (40.7)	115 (40.9)
Degarelix*	20 (0.6)	27 (1.3)	8 (1.2)	28 (1.4)	19 (1.8)	10 (1.7)	5 (1.8)
Hormonal agents	**107 (3)**	**845 (41)**	**470 (69.3)**	**1377 (66.9)**	**646 (61.9)**	**284 (49.2)**	**251 (89.3)**
First Generation Hormonal Agents	**107 (3)**	**86 (4.2)**	**18 (2.7)**	**228 (11.1)**	**128 (12.3)**	**83 (14.4)**	**35 (12.5)**
Flutamide	15 (0.4)	31 (1.5)	7 (1)	66 (3.2)	41 (3.9)	24 (4.2)	12 (4.3)
Nilutamide	0 (0)	16 (0.8)	5 (0.7)	53 (2.6)	29 (2.8)	17 (2.9)	12 (4.3)
Bicalutamide	92 (2.6)	39 (1.9)	6 (0.9)	109 (5.3)	58 (5.6)	42 (7.3)	11 (3.9)
ARPI	**0 (0)**	**759 (36.8)**	**452 (66.7)**	**1149 (55.9)**	**518 (49.7)**	**201 (34.8)**	**216 (77.0)**
Abiraterone	0 (0)	450 (21.8)	0 (0)	644 (31.3)	220 (21.1)	106 (18.4)	103 (36.7)
Enzalutamide	0 (0)	121 (5.9)	223 (32.9)	505 (24.6)	298 (28.6)	95 (16.5)	113 (40.2)
Apalutamide	0 (0)	188 (9.1)	144 (21.2)	0 (0)	0 (0)	0 (0)	0 (0)
Darolutamide	0 (0)	0 (0)	85 (12.5)	0 (0)	0 (0)	0 (0)	0 (0)
Taxanes	**0 (0)**	**1080 (52.4)**	**1 (0.1)**	**595 (28.9)**	**389 (37.3)**	**259 (44.9)**	**234 (83.3)**
Docetaxel	0 (0)	1080 (52.4)	1 (0.1)	536 (26.1)	244 (23.4)	145 (25.1)	100 (35.6)
Cabazitaxel	0 (0)	0 (0)	0 (0)	59 (2.9)	145 (13.9)	114 (19.8)	134 (47.7)
iPARPs	0 (0)	0 (0)	0 (0)	0 (0)	0 (0)	0 (0)	0 (0)
Olaparib	0 (0)	0 (0)	0 (0)	0 (0)	0 (0)	0 (0)	0 (0)
Niraparib	0 (0)	0 (0)	0 (0)	0 (0)	0 (0)	0 (0)	0 (0)
Rucaparib	0 (0)	0 (0)	0 (0)	0 (0)	0 (0)	0 (0)	0 (0)
Talazoparib	0 (0)	0 (0)	0 (0)	0 (0)	0 (0)	0 (0)	0 (0)
Radiopharmaceuticals							
Radium–223 (^{223}Ra)	0 (0)	0 (0)	0 (0)	37 (1.8)	38 (3.6)	47 (8.1)	40 (14.2)

*These drugs are usually maintained as part of the ADT combinations despite disease progression.
†Prostatectomy and TURP.
ADT, androgen deprivation therapy; ARPI, androgen receptor pathway inhibitors; iPARPs, poly ADP ribose polymerase inhibitors; MDR, M#, metastasis o no metastasis; N#/, compromised or not lymph nodes; mHSPC, metastatic hormone–sensitive prostate cancer; nmCRPC, non–metastatic castration–resistant prostate cancer; mCRPC, metastatic castration–resistant prostate cancer; PC, prostate cancer. metastatic directed radiotherapy; TURP, transurethral resection of the prostate.

Table S7. Duration of prostate cancer treatments during the follow–up period according to disease stage.

**Days, mean (*SD*)**	**PC (N1/M0) n=3,560**	**mHSPC n=2,062**	**nmCRPC n=678**	**mCRPC L1 n=2,057**	**mCRPC L2 n=1,043**	**mCRPC L3 n=577**	**mCRPC L4+ n=281**
ADT	**767.6 (615.4)**	**523.7 (264.5)**	**702.1 (301.5)**	**188.2 (87)**	**161.8 (39.3)**	**138.5 (56.3)**	**158.3 (79.8)**
Buserelin*	617.2 (157)	546 (300.4)	645 (302.9)	193.8 (87.7)	145.3 (45)	137.1 (55.6)	196 (90)
Leuprorelin*	814.9 (692.4)	522 (264.3)	715.2 (304.9)	195.7 (87.8)	161.5 (39.5)	138.8 (60.2)	160.4 (82)
Goserelin*	731.6 (469.9)	552.2 (272.7)	675.7 (284.3)	178.5 (82.4)	162.2 (37.2)	147.6 (55.5)	166.2 (69.6)
Triptorelin*	720.5 (543.6)	516.9 (259.5)	703.1 (298.7)	183.1 (86.9)	163.4 (39.3)	135.8 (53.4)	151.9 (81.3)
Degarelix*	1001.6 (796.3)	496.6 (285.3)	627.1 (458.7)	174.9 (83.8)	156.3 (34)	128.8 (31.9)	153 (46.5)
Hormonal agents	**674.6 (120.5)**	**532.4 (268.7)**	**713.9 (303.6)**	**187.1 (73.8)**	**166.1 (34.3)**	**152.3 (53.7)**	**159.1 (81)**
First Generation Hormonal Agents	**674.6 (120.5)**	**538.4 (263.4)**	**684.5 (278.2)**	**177 (79.2)**	**153.2 (47.5)**	**172.3 (68.5)**	**168.2 (87.6)**
Flutamide	641.8 (78.1)	481.1 (255.9)	740.4 (124.1)	167.3 (71.5)	151.8 (50.7)	180.9 (65.2)	145.8 (63)
Nilutamide	NA	620.6 (336)	769.9 (425)	159.2 (69)	156.9 (45.8)	157.1 (61.9)	192.8 (93.2)
Bicalutamide	679.9 (125.6)	560.3 (230)	548.3 (257.3)	191.5 (86.1)	151.4 (46.6)	173.5 (73.2)	165.7 (104.2)
ARPI	**NA**	**531.3 (269.8)**	**715.1 (304.7)**	**189.1 (72.6)**	**172.2 (23.6)**	**144.1 (43.9)**	**157.7 (80)**
Abiraterone	NA	533.4 (276.7)	NA	190 (73.4)	171.7 (24.1)	140.9 (41.4)	153.2 (75.6)
Enzalutamide	NA	512.2 (248.5)	706 (316.6)	188 (71.6)	172.6 (23.3)	147.6 (46.6)	178.3 (81.7)
Apalutamide	NA	538.4 (266.9)	724.1 (294.2)	NA	NA	NA	NA
Darolutamide	NA	NA	723.6 (293.1)	NA	NA	NA	NA
Taxanes	NA	**520.2 (259.7)**	**530.2 (0)**	**148.2 (88.5)**	**144.3 (52.4)**	**122.4 (56.7)**	**162.9 (80.5)**
Docetaxel	NA	520.2 (259.7)	530.2 (0)	155.2 (87)	169.8 (23.5)	149.3 (51.7)	174.3 (80)
Cabazitaxel	NA	NA	NA	84.5 (77.2)	101.4 (59.3)	88.3 (42.8)	182.5 (77.5)
iPARPs	NA	NA	NA	NA	NA	NA	NA
Olaparib	NA	NA	NA	NA	NA	NA	NA
Niraparib	NA	NA	NA	NA	NA	NA	NA
Rucaparib	NA	NA	NA	NA	NA	NA	NA
Talazoparib	NA	NA	NA	NA	NA	NA	NA
Radiopharmaceuticals							
^{223}Ra	NA	NA	NA	157.3 (76.9)	171.8 (23.3)	155.5 (69)	180.1 (78.8)
**Days, median (P25–P75)**							
Androgen deprivation therapy (ADT)	677 (523–745)	364.7 (304.5–730)	705.2 (435.7–936.2)	155.5 (127–273)	168 (153–183)	129 (111–149)	150 (100–214)
Buserelin*	693 (557–713.5)	364.2 (281.9. 774.5)	610 (335.8. 864.7)	164.3 (129.4–255.5)	161 (134.8–175.5)	130.5 (117.8–146.8)	182 (155.5–249)
Leuprorelin*	673 (515–752)	364.9 (300.9–733.5)	722.2 (441.9–942.7)	158 (132.5–283)	168 (152.5–183)	126 (109.3–148.8)	149.5 (99.5–220.3)
Goserelin*	658 (613.5–718)	514.1 (304.5–761)	727.7 (388.4–889.1)	152 (124.3–254.5)	167.5 (153.5–180)	136 (118.3–156.5)	162 (132.5–209.5)
Triptorelin*	682 (500–746.3)	361.3 (308.7–706.1)	699.8 (434.8–953)	152.5 (121–264)	169 (155–186)	128 (111–148.5)	147 (94–203.5)
Degarelix*	684 (670.8–933)	339.7 (308.9–715)	584.5 (188.3–1053.8)	146.3 (122.4–179.8)	162 (155.5–168)	131 (110.8–147.5)	149 (126–154)
Hormonal agents	682 (644.5–741.5)	392.6 (307.8–736)	725.4 (437.8–947.3)	156.5 (138.5–242)	169 (156–185)	135 (121.8–157.3)	**150 (99–220)**
First Generation Hormonal Agents	682 (644.5–741.5)	469.8 (309.1–761.8)	676.8 (518.2–840)	150.5 (129.8–195)	164 (149–179.8)	146 (123.5–213.5)	152 (97.5–239.5)
Flutamide	642 (612–678)	340.6 (296.2–670.5)	733.7 (676.8–824.2)	147.8 (130.6–174.3)	164 (150–177.3)	168 (123.5–223.3)	151 (92.8–170.3)
Nilutamide	NA	659.9 (338.2–821.6)	637.8 (509.9–1123.3)	140 (125.3–164.5)	165.5 (149.8–185.5)	134 (124–184)	164.5 (124.5–288)
Bicalutamide	691 (653–747)	606.3 (309.7–771.8)	483.7 (353.1–737.2)	160.5 (133–272)	164 (146–179.5)	145.5 (124–226.8)	160 (63–249)
ARPI	NA	367.9 (307.8–727.4)	725.7 (437.1–950.9)	157.5 (139.5–245)	171 (159–186)	133 (121–149)	149 (99.5–214.3)
Abiraterone	NA	366.7 (307.9–718.7)	NA	158.5 (139–248.3)	170 (159–185)	132 (119.3–146.8)	145 (100–198)
Enzalutamide	NA	410.9 (300.8–700.1)	706.4 (411.9–951.6)	156.5 (140–242)	171 (159.3–186.8)	134 (122.5–151.5)	170 (119.3–250.5)
Apalutamide	NA	448.9 (315.1–761.9)	745 (446.6–937.9)	NA	NA	NA	NA

Days, mean (*SD*)	PC (N1/M0) n=3,560	mHSPC n=2,062	nmCRPC n=678	mCRPC L1 n=2,057	mCRPC L2 n=1,043	mCRPC L3 n=577	mCRPC L4+ n=281
Darolutamide	NA	NA	698.8 (466.3–955.3)	NA	NA	NA	NA
Taxanes	NA	362.7 (306.6–728.9)	530.2 (530.2–530.2)	108.3 (89.7–187.5)	164 (107–177)	118 (92–140)	152.5 (105.8–225)
Docetaxel	NA	362.7 (306.6–728.9)	530.2 (530.2–530.2)	111.7 (93.6–204.4)	169 (157–181)	135 (119–155)	163 (126–240)
Cabazitaxel	NA	NA	NA	63 (45–90.5)	87.5 (48.5–165)	85 (62.3–102)	166 (134–249)
iPARPs	NA	NA	NA	NA	NA	NA	NA
Olaparib	NA	NA	NA	NA	NA	NA	NA
Niraparib	NA	NA	NA	NA	NA	NA	NA
Rucaparib	NA	NA	NA	NA	NA	NA	NA
Talazoparib	NA	NA	NA	NA	NA	NA	NA
Radiopharmaceuticals							
^{223}Ra	NA	NA	NA	124.7 (100.2–193.8)	173.5 (152.3–187.8)	132 (124–151)	166 (129.3–252.8)

ADP, Androgen deprivation therapy; ARPI, androgen receptor pathway inhibitors; iPARPs, inhibitors of the enzyme poly–ADP ribose polymerase; ^{223}Ra, Radium–223; M#, metastasis o no metastasis; N#/, compromised or not lymph nodes; mHSPC, metastatic hormone–sensitive prostate cancer; nmCRPC, non–metastatic castration–resistant prostate cancer; mCRPC, metastatic castration–resistant prostate cancer; PC, prostate cancer.

Table S8. Concomitant medication and procedures during the follow–up period.

	Locally advanced PC (N1/M0)	mHSPC	nmCRPC	mCRPC L1	mCRPC L2	mCRPC L3	mCRPC L4+
**N (%)**	3560 (100)	2062 (100)	678 (100)	**2057 (100)**	**1043 (100)**	**577 (100)**	**281 (100)**
Medications							
Analgesic & Pain Needs (ATC code: N02)	2384 (67.0)	1775 (86.1)	530 (78.2)	1773 (86.2)	874 (83.8)	477 (82.7)	226 (80.4)
Antiresorptive bone agents (ATC code: M5B)	285 (8.0)	536 (26.0)	55 (8.1)	663 (32.2)	313 (30.0)	169 (29.3)	86 (30.6)
Procedures							
Blood/ other transfusions	43 (1.2)	37 (1.8)	6 (0.9)	64 (3.1)	25 (2.4)	11 (1.9)	4 (1.4)

M#, metastasis o no metastasis; N#/, compromised or not lymph nodes; mHSPC, metastatic hormone–sensitive prostate cancer; nmCRPC, non–metastatic castration–resistant prostate cancer; mCRPC, metastatic castration–resistant prostate cancer; PC, prostate cancer.

Supplementary Figures

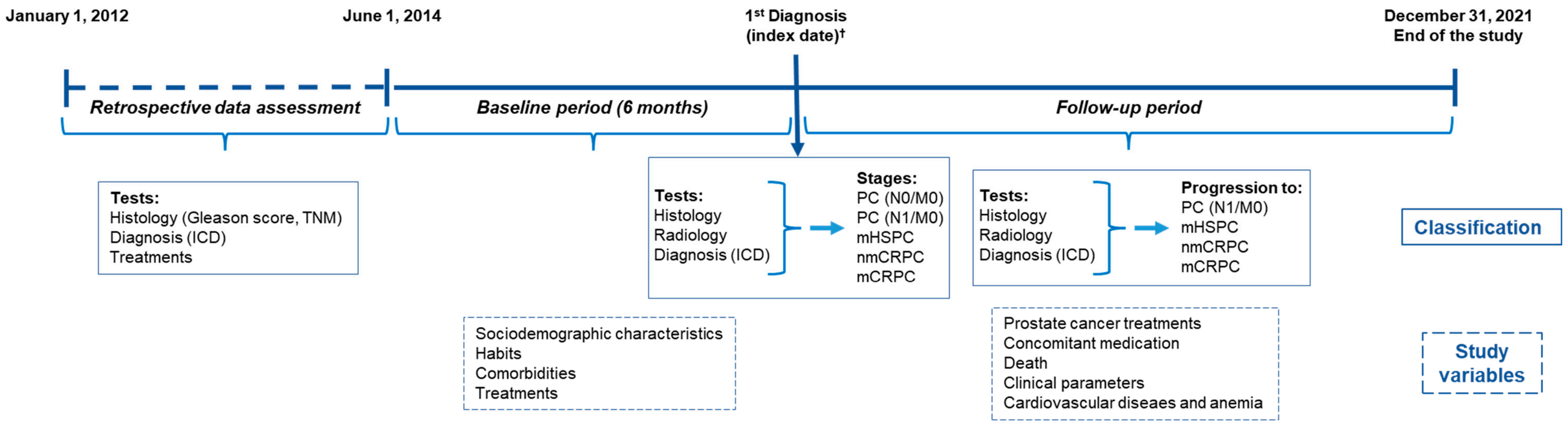

Figure S1. Diagram of the study design. †Index date would be any date between June 1, 2014 and December 31, 2021; ICD, International Classification of Diseases; M#, metastasis o no metastasis; N#/, compromised or not lymph nodes; mHSPC, metastatic hormone–sensitive prostate cancer; nmCRPC, non–metastatic castration–resistant prostate cancer; mCRPC, metastatic castration–resistant prostate cancer; PC, prostate cancer; TNM, tumor, node and metastasis.

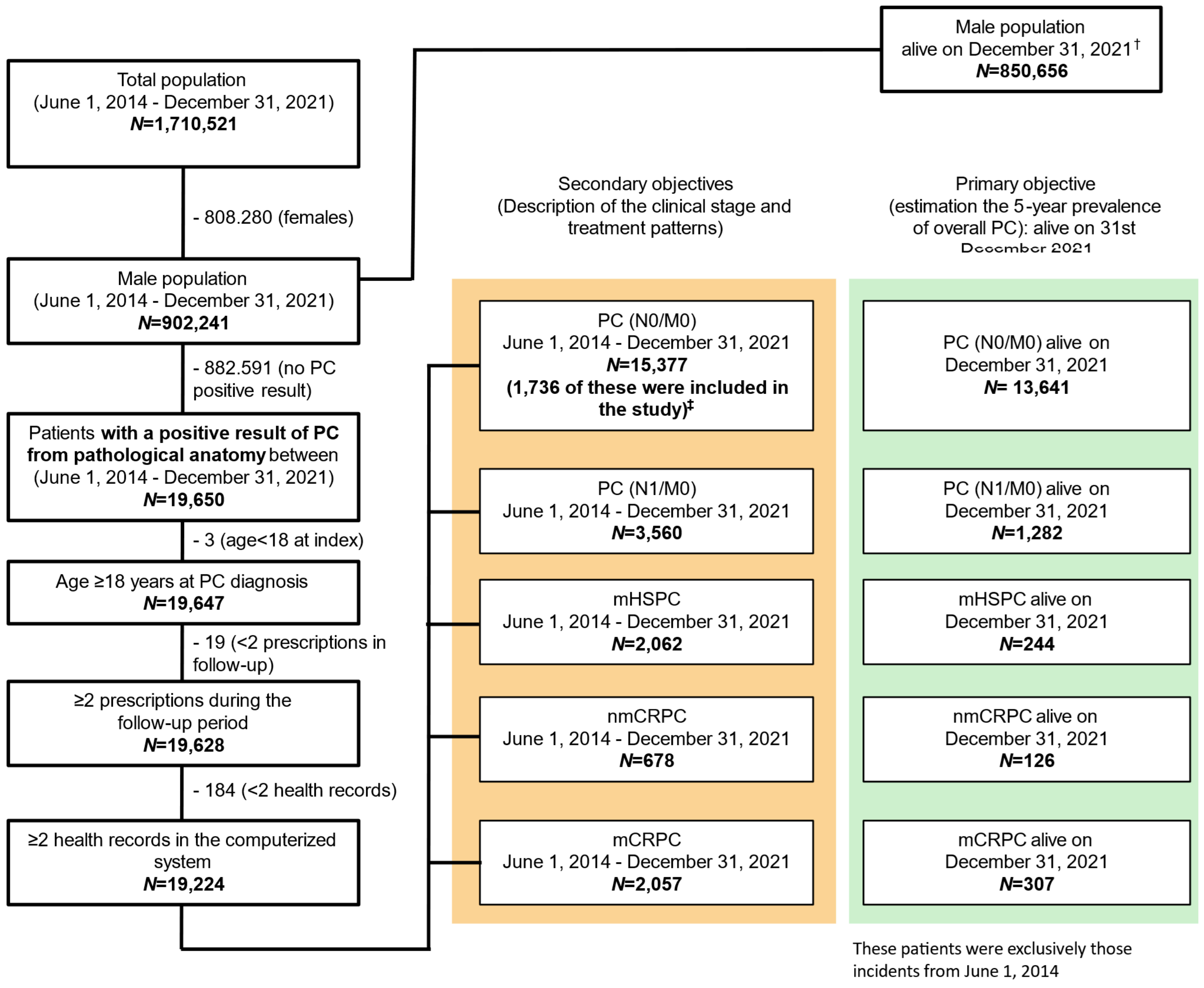

Figure S2. Patient selection flow chart and classification into study cohorts. †This population contains all males comprised in BIG–PAC® database on December 31, 2021, regardless of the inclusion criteria, ‡These patients were included as they progressed to further stages and had a PC positive result within June 2014–December 2021

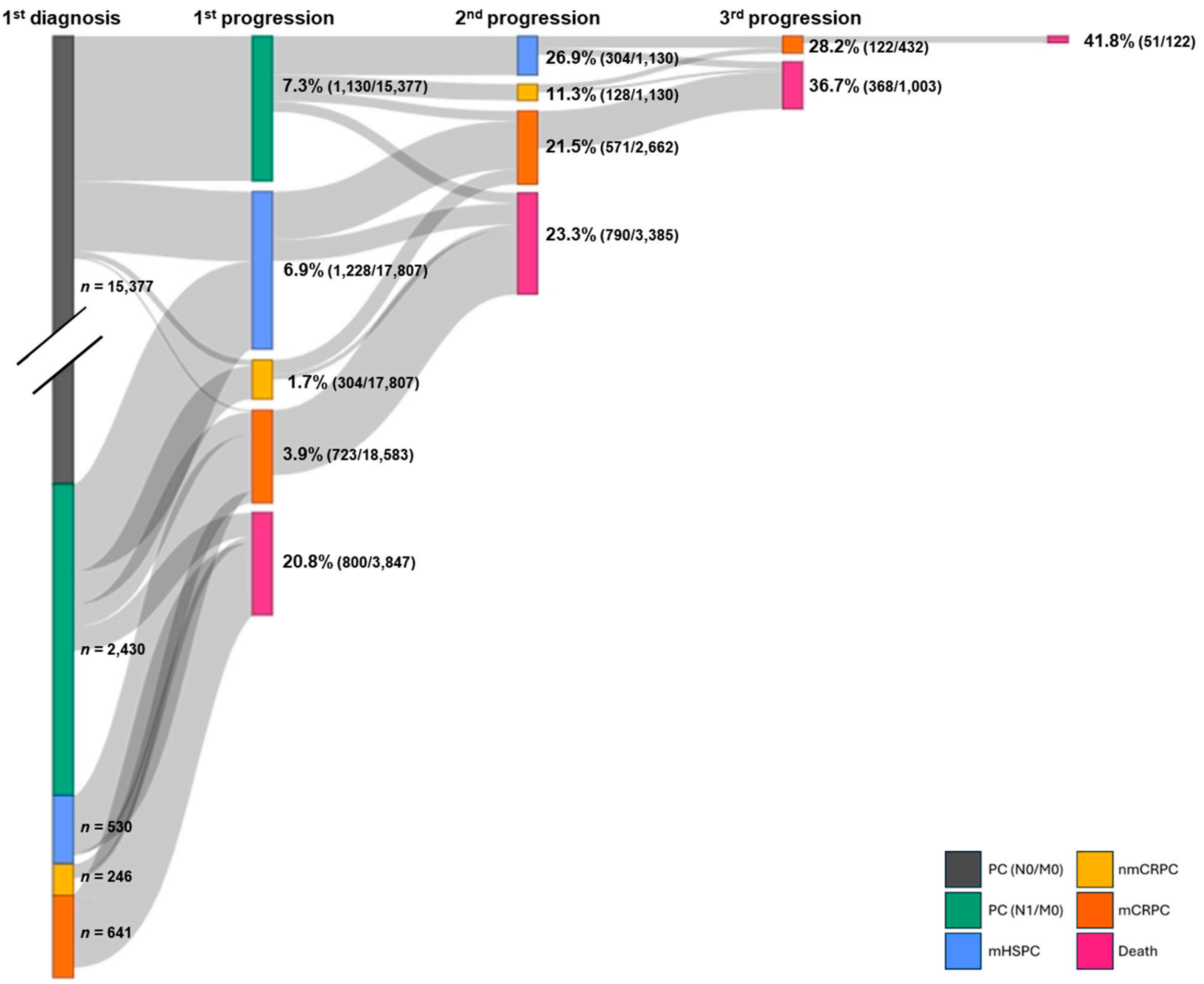

Figure S3. Flow of unique patients from the moment they entered the study until the end of the study or follow–up. Recorded death of patients was all–caused death, not cancer-specific. To simplify the graph, we added an axis break in the localized PC (N0M0) patients who did not progress (13,641 patients). Cohorts are represented in each column according to stage severity (less severe on the top). Denominators are the sum of patients from the origin stage, i.e., mHSPC patients in the first progression come from the PC (N0/M0) and PC (N1/M0) populations, hence their denominator is the sum of those two populations.

	2014	2015	2016	2017	2018	2019	2020	2021
**Surgery**	PC(N1/M0)	PC(N1/M0)	PC(N1/M0)	PC(N1/M0)	PC(N1/M0)	PC(N1/M0)	PC(N1/M0)	PC(N1/M0)
**Radiotherapy**	PC(N1/M0)	PC(N1/M0)	PC(N1/M0)	PC(N1/M0)	PC(N1/M0)	PC(N1/M0)	PC(N1/M0)	PC(N1/M0)
**ADT**	PC(N1/M0), nmCRPC, mHSPC, mCRPC	PC(N1/M0), nmCRPC, mHSPC, mCRPC	PC(N1/M0), nmCRPC, mHSPC, mCRPC	PC(N1/M0), nmCRPC, mHSPC, mCRPC	PC(N1/M0), nmCRPC, mHSPC, mCRPC	PC(N1/M0), nmCRPC, mHSPC, mCRPC	PC(N1/M0), nmCRPC, mHSPC, mCRPC	PC(N1/M0), nmCRPC, mHSPC, mCRPC
**1st generation hormonal agents**	PC(N1/M0), nmCRPC, mHSPC, mCRPC	PC(N1/M0), nmCRPC, mHSPC, mCRPC	PC(N1/M0), nmCRPC, mHSPC, mCRPC	PC(N1/M0), nmCRPC, mHSPC, mCRPC	PC(N1/M0), nmCRPC, mHSPC, mCRPC	PC(N1/M0), nmCRPC, mHSPC, mCRPC	PC(N1/M0), nmCRPC, mHSPC, mCRPC	PC(N1/M0), nmCRPC, mHSPC, mCRPC
**Taxanes**	mCRPC	mCRPC	mCRPC	mCRPC	mCRPC	mHSPC, mCRPC	mHSPC, mCRPC	mHSPC, mCRPC
Docetaxel	mCRPC	mCRPC	mCRPC	mCRPC	mCRPC	mHSPC, mCRPC	mHSPC, mCRPC	mHSPC, mCRPC
Cabazitaxel	mCRPC	mCRPC	mCRPC	mCRPC	mCRPC	mCRPC	mCRPC	mCRPC
**ARPI**	mCRPC	mCRPC	mCRPC	mCRPC	mCRPC	mCRPC	mHSPC, mCRPC	nmCRPC, mHSPC, mCRPC
Enzalutamide		mCRPC	mCRPC	mCRPC	mCRPC	mCRPC	mCRPC	nmCRPC, mCRPC
Abiraterone							mHSPC	mHSPC
Apalutamide								mHSPC, nmCRPC
Darolutamide								nmCRPC
**223-Ra**		mCRPC	mCRPC	mCRPC	mCRPC	mCRPC	mCRPC	mCRPC

Figure S4. Therapeutic positioning reports/technical sheets or clinical guidelines followed in Spain between 2014 and 2021. Funding does not necessarily correspond to the date of positive opinion. ADT, androgen deprivation therapy; ARPI, androgen receptor pathway inhibitor; mCRPC, metastatic castration-resistant prostate cancer; mHSPC, metastatic hormone-sensitive prostate cancer; PC, prostate cancer; PC (N0/M0), prostate cancer without nodal or distant metastases; Ra-223, radium-223 dichloride.

Sources of information:

Surgery and radiotherapy:

EAU Guidelines on Prostate Cancer (https://uroweb.org/guidelines/archive/prostate-cancer). Of note, between 2010 and 2021 there was no regular update in the national guidelines, which is why EAU guidelines were followed.

ADT: buserelina: https://cima.aemps.es/cima/dochtml/ft/57011/FT_57011.html , leuprorelina: https://cima.aemps.es/cima/dochtml/ft/74980/FichaTecnica_74980.html , goserelina: https://cima.aemps.es/cima/dochtml/ft/61367/ft_61367.html , triptorelina: https://cima.aemps.es/cima/dochtml/ft/58404/FichaTecnica_58404.html , degarelix https://cima.aemps.es/cima/dochtml/ft/08504001/FT_08504001.html.

HORMONAL AGENTS: flutamide: https://cima.aemps.es/cima/dochtml/ft/62912/FichaTecnica_62912.html , bicalutamide https://cima.aemps.es/cima/dochtml/ft/68878/FichaTecnica_68878.html .

TAXANES: docetaxel (https://cima.aemps.es/cima/pdfs/es/ft/72635/72635_ft.pdf; https://www.aemps.gob.es/informa/boletines-aemps/boletin-mensual-de-la-aemps-sobre-medicamentos-de-uso-humano-del-mes-de-septiembre-de-2019/ , cabazitaxel: https://www.aemps.gob.es/informa/boletines-aemps/boletinMensual/2011/enero/informe-enero-7/ https://www.sanidad.gob.es/profesionales/medicamentos.do?metodo=verDetalle&cn=677657; https://cima.aemps.es/cima/pdfs/es/ft/85714/85714_ft.pdf)

ARPIS: enzalutamide: https://www.aemps.gob.es/medicamentosUsoHumano/informesPublicos/docs/IPT-enzalutamida-Xtandi.pdf , https://www.aemps.gob.es/medicamentosUsoHumano/informesPublicos/docs/2021/IPT_31-2021-Xtandi2.pdf , abiraterone: https://www.aemps.gob.es/medicamentosUsoHumano/informesPublicos/docs/IPT_8-2020-abiraterona-Zytig.pdf , apalutamide: https://www.aemps.gob.es/medicamentos-de-uso-humano/informes-de-posicionamiento-terapeutico/ , darolutamide: https://www.aemps.gob.es/informa/informes-de-posicionamiento-terapeutico/informe-de-posicionamiento-terapeutico-de-darolutamida-nubeqa-en-cancer-de-prostata-resistente-a-la-castracion-no-metastasico/

223-Ra: https://www.sanidad.gob.es/profesionales/medicamentos.do?metodo=verDetalle&cn=700919 , https://cima.aemps.es/cima/dochtml/ft/113873001/FT_113873001.html .
